# Housing after redevelopment: Where and to what buildings do displaced residents move?

**DOI:** 10.1177/00420980251376156

**Published:** 2025-10-20

**Authors:** Fiona Kauer, Elena Lutz, David Kaufmann

**Affiliations:** ETH Zurich, Institute for Spatial and Landscape Development IRL, Switzerland; ETH Zurich, Institute for Spatial and Landscape Development IRL, Switzerland; ETH Zurich, Institute for Spatial and Landscape Development IRL, Switzerland

**Keywords:** densification, displacement, gentrification, housing insecurity, urban redevelopment, 致密化, 流离失所, 绅士化, 住房无保障, 城市再开发

## Abstract

Urban redevelopment often involves the demolition or renovation of the existing housing stock, which can result in the direct displacement of residents. We examine how tenure type, defined as living in for-profit versus non-profit housing, affects the location and the housing characteristics of displaced residents after displacement. Using individual-level data of all residents in the Zurich region of Switzerland, we observe the direct displacement of 12,599 residents between 2016 and 2020. First, we descriptively analyze who is directly displaced and compare their housing situation before and after relocation. Displacement is socially stratified and predominantly affects low-income residents, earning on average only 69.7% of the median income of all movers. Residents without Swiss citizenship were substantially more likely to be displaced (9.6 percentage points). After their displacement, residents consumed more floor space than before. Next, we use propensity score matching to compare displaced residents from for-profit and displaced residents from non-profit housing with a respective comparison group of socio-economically similar individuals who moved for reasons other than building demolition or renovation. We find that displaced residents from for-profit projects tend to move shorter distances and to lower-income areas. They consume less floor space and are slightly more likely to move to post-war buildings (1945–1970). These inequalities in moving patterns are less prevalent for residents from non-profit redevelopment projects. This shows that assistance in finding housing after displacement and non-profit housing more generally can mitigate some of the negative aspects of displacement.

## 摘要

城市再开发通常涉及拆除或翻新现有住房，可能导致居民直接流离失所。我们考察了住房权属类型（定义为居住于营利性住房还是非营利性住房）对流离失所的居民在搬迁后所处的区位及其住房特征产生的影响。利用瑞士苏黎世地区所有居民的个体层面数据，我们观察到 2016 年至 2020 年间有 12,599 名居民直接流离失所。首先，我们对直接流离失所的居民进行描述性分析，并比较其搬迁前后的住房状况。流离失所具有社会分层性，主要影响低收入居民，他们的平均收入仅为所有搬迁者中位数收入的 69.7%。没有瑞士公民身份的居民流离失所的可能性要大得多（高 9.6 个百分点）。居民在搬迁后居住的建筑面积比搬迁前更大。接下来，我们使用倾向评分匹配法，将从营利性住房搬迁的居民和从非营利性住房搬迁的居民，分别与一组社会经济特征相似、且因建筑拆除或翻新以外原因迁居的对照组人群进行对比。我们发现，在营利性项目中被搬迁的居民往往会迁居到距离较近且收入较低的地区。他们居住的建筑面积更小，并且更有可能迁入战后建筑（1945-1970 年）。对于非营利性再开发项目的居民来说，这些迁居模式的不平等现象并不那么普遍。这表明，为流离失所居民提供找房援助，以及更普遍地提供非营利性住房，可缓解搬迁带来的一些负面影响。

## Introduction

Increased urban redevelopment activities raise concerns about the displacement of residents. Indeed, the demolition and replacement or renovation of old buildings forces residents to leave their homes because their rental contracts are terminated. Often, they cannot afford the rent of the new apartments (Baeten et al., 2017; Debrunner et al., 2024). Since these residents got evicted, their relocation can be classified as direct displacement (Marcuse, 1985; Slater, 2009). Displacement may negatively impact physical and mental health (Gillespie et al., 2021), or result in long-distance moves, separating residents from their existing schools, workplaces, and social environments (Reades et al., 2023).

Urban redevelopment and gentrification-induced displacement are widely studied in urban research (Freeman and Braconi, 2004; Gehriger, 2024; Hochstenbach and Musterd, 2018; Lutz et al., 2024; Meuth and Reutlinger, 2023; Ramiller, 2022; Raymond et al., 2021). Some recent studies have used individual-level data to quantify where and to what neighborhoods residents move after housing demolition (Chyn, 2018; Miltenburg et al., 2018; Reades et al., 2023). This is an important question as it helps to understand the individual-level repercussions of displacement. However, these studies focus solely on either the non-profit sector, such as the renewal of public housing, or on gentrifying neighborhoods. In general, existing research lacks data to distinguish displacement by tenure type between non-profit and for-profit housing (Elliott-Cooper et al., 2020). This distinction is important because non-profit housing providers often assist their residents to find housing or provide them with an alternative property.

The existing literature finds that displaced residents from public housing who received financial assistance moved to higher-income neighborhoods in the USA (Chyn, 2018) and in the Netherlands (Miltenburg et al., 2018). In contrast, low-income residents moving from gentrifying neighborhoods without assistance moved to suburban areas in the Netherlands (Hochstenbach and Musterd, 2018), and in the USA, such residents moved to slightly lower-income neighborhoods but did not move far (Freeman et al., 2024). These findings point to the importance of the type of tenure, and the need to differentiate between for-profit and non-profit when studying displacement.

Furthermore, Sims and Sarmiento (2023) argue for studying displacement not only as a spatial event but also for how it affects the housing situation of residents. Simultaneously studying the neighborhood *and* the characteristics of the housing unit to which displaced individuals move enhances our understanding of the impact of displacement, as displaced individuals may face material constraints and difficult decisions between long-distance moves or relocating to lower-quality housing to stay close to their social networks. Consequently, instead of long-distance moves, displacement might lead to other localized forms of inequality and exclusion.

This article addresses these gaps and contributes to the literature by comparing the relocation patterns of displaced residents from for-profit and non-profit redevelopment projects. Additionally, our joint focus on neighborhood and housing characteristics provides insights into the impacts of displacement on residents beyond the spatial consequences. We ask: *Where do directly displaced residents move? What type of housing do they move to? And how do these patterns differ between for-profit and non-profit redevelopments?*

We investigate direct displacement in the Zurich region, which is Switzerland’s largest urban area. With the revision of the Federal Spatial Planning Act in 2014, Switzerland introduced densification as a legal planning goal (SPA, 2014). Hence, there have been increasing numbers of housing demolitions and higher-density replacement constructions (Büchler and Lutz, 2024; Kauer et al., 2025; Nebel et al., 2017), leading to the direct displacement of tenants (Gehriger, 2024; Lutz et al., 2024; Meuth and Reutlinger, 2023). The Zurich region has both strong for-profit and non-profit actors in the housing market. Non-profit actors include housing cooperatives, foundations, and associations, and most of them are guided by public interest under the charter of non-profit housing (BWO, 2024). Housing cooperatives provide the largest stock of non-profit housing. Tenants are usually members of the cooperatives. Cooperatives support tenants in the event of redevelopment, typically by providing an alternative apartment. In contrast, residents displaced from for-profit projects usually have to find new housing independently.

We use geo-coded individual-level yearly panel data on all 1.5 million residents in the Zurich region from 2012 to 2022. This fine-grained data allows us to precisely analyze the relocation patterns of directly displaced residents. In a first step, we look at relocation patterns descriptively and compare displaced residents’ housing and neighborhood situation before and after relocation. Second, we use propensity score matching to identify two comparison groups of residents living in either for-profit or non-profit housing who have similar characteristics to those directly displaced but moved for reasons other than demolition or renovation. This allows us to compare residents with similar resources for finding and moving to a new apartment. We then run regression analyses to answer our research questions.

We find that between 2016 and 2020, 2043 multi-family buildings were demolished or fully renovated, leading to the direct displacement of 12,599 residents, that is, 0.8% of all residents living in the Zurich region. Redevelopment-induced displacement is socially stratified and predominantly affects low-income residents. Non-Swiss citizens are also more likely to be affected. Rather than displacing residents from the Zurich city center neighborhoods to the suburbs, displacement leads to moves between peripheral urban neighborhoods and suburban areas. On average, displaced residents from for-profit redevelopment projects move short distances and relocate to low-income neighborhoods, which is in line with previous research (Freeman et al., 2024; Reades et al., 2023). In contrast, we do not find a significant association between moving distance or neighborhood income level and being displaced from non-profit redevelopment projects. In terms of housing characteristics before and after displacement, we find that, on average, displaced residents move to newer buildings and consume more floor space. Yet, compared to similar residents who moved for reasons other than redevelopment, displaced residents from for-profit and non-profit projects are more likely to move to smaller housing units. Additionally, those displaced from for-profit sites are slightly more likely to move to older apartments. Thus, inequalities in relocation patterns are more pronounced for residents displaced from profit-oriented redevelopment projects than from non-profit projects when compared to the comparison groups.

We begin this article with a literature review on displacement and outline our theoretical expectations, followed by a section on urban redevelopment in the Zurich region. We then proceed with a section on methods. In the Results section, we discuss descriptive findings and the regression results. In the Discussion and conclusion section, we highlight the main findings and outline potential avenues for future research.

## Literature review

According to the influential definition by Marcuse (1985), displacement describes involuntary moves of residents due to conditions affecting their housing unit or neighborhood. Scholars differentiate between direct and indirect forms of displacement (Elliott-Cooper et al., 2020; Marcuse, 1985; Slater, 2009, 2021). This article focuses on direct displacement, namely when residents move because staying in their apartment is impossible due to external forces (Slater, 2009), in our case, when a building is demolished or renovated. Direct displacement often affects low-income residents, who cannot afford to return to the redeveloped sites (Elliott-Cooper et al., 2020). Additionally, redevelopment projects are often preceded by prolonged periods of decline (Sakizlioglu and Uitermark, 2014). This decline can be seen as a strategy by developers to facilitate the redevelopment process by gradually pressuring tenants to leave their apartments. As a result, tenants face insecure housing situations over a long period or move out long before redevelopment begins. These early relocations are a frequently overlooked form of displacement.

Research on the relocation of displaced residents from council estates in London, UK (Reades et al., 2023), or those moving from gentrifying neighborhoods in the USA (Freeman et al., 2024), found that residents moved short distances. In contrast, Hochstenbach and Musterd (2018) showed that low-income residents in the Netherlands increasingly moved away from gentrifying city center neighborhoods toward suburban areas and that those movements increased during times of urban growth (Booi, 2024), a trend the authors refer to as the suburbanization of poverty (Hochstenbach and Musterd, 2018). Banabak et al. (2024) contrast this finding with movement patterns in Vienna, Austria. In Vienna’s highly regulated and tenure-segmented housing market, they find that low-income residents tend to move short distances and stay within the gentrifying city center but show high movement frequency. They argue that spatial patterns of where residents move depend on how accessible a specific area or segment of the housing market is.

Instead of examining the distance between old and new homes, a large body of literature has been investigating the quality of the neighborhood into which displaced residents move (Chyn, 2018; Miltenburg et al., 2018; Reades et al., 2023). It is important to note that these studies focus on the demolition of public housing and on the rehousing of tenants. Such renewal strategies are usually implemented to combat segregation and counteract negative neighborhood effects (Miltenburg et al., 2018). Programs such as the Moving to Opportunity experiment in the USA randomly distributed housing vouchers among public housing tenants who agreed to participate in the experiment (Chyn, 2018). These vouchers could be used to access higher-income neighborhoods. Similarly, social housing renewal programs in the Netherlands provided relocation counseling, financial compensation, and priority in social housing access to those displaced (Miltenburg et al., 2018). The results of such studies showed that assistance allowed displaced residents to move to higher-income neighborhoods (Chyn, 2018; Miltenburg et al., 2018). In contrast, people moving from renewal sites of council estates in the UK relocated to lower-income neighborhoods (Reades et al., 2023). When focusing on moving patterns in gentrifying neighborhoods in the USA, Freeman et al. (2024) observed the same patterns. In short, whether residents can access higher-income neighborhoods or move to poorer areas likely depends on the relocation assistance they receive.

Taken together, these findings indicate that relocation patterns differ based on tenure type and how redevelopment is implemented, which typically differs between for-profit and non-profit housing. As for-profit investors redevelop a site to generate profits, it is unlikely that they actively support residents to stay in the redeveloped building or move to an alternative site (see e.g. Debrunner et al., 2024). In contrast, non-profit housing is organized by the state or by housing cooperatives. In housing cooperatives, tenants are usually members of the cooperative, meaning that they collectively manage their own housing (Larsen, 2024). The shared ownership and the decommodification of housing contributes to a redevelopment approach where tenants have a say in how projects and rehousing are implemented.

Recent research has criticized the reduction of gentrification-induced displacement to a spatial event (Sims and Sarmiento, 2023). Focusing exclusively on where displaced residents move to might not be enough. In the case of the Netherlands, Hochstenbach and Musterd (2018) hypothesize that residents might be able to remain within the city center if they move together with other households or to smaller apartments, potentially leading to overcrowding. Similarly, Sims and Sarmiento (2023) analyzed survey data and found high levels of overcrowding, housing insecurity, and poor housing conditions among displaced residents in Santa Ana, California. Moreover, displacement might result in insecure housing tenure, for example when displaced individuals move to old buildings with a higher likelihood of being demolished soon. As Banabak et al. (2024) show in the case of Vienna, low-income residents move a lot within gentrifying areas in the urban center, which they see as a sign of low-income residents being trapped in insecure housing conditions. For displaced residents, such a situation increases the risk of having to move again. Consequently, for a better understanding of individual-level impacts of direct displacement, both *where* displaced residents move to and to *what* housing they relocate matter and need to be studied together. In this article, we contribute to the debate on displacement by focusing on the differences between two tenure types, namely for-profit and non-profit redevelopment, and where residents move to but also what type of housing they move to. In light of previous studies, we hypothesize the following:

**H1:** *Displaced residents from for-profit housing move short distances but are likely to move to low-income neighborhoods.*
**H2:** *Displaced residents from for-profit housing move to small apartments and old housing stock.*
**H3:** *Displaced residents from non-profit housing move short distances and are less likely to move to small or old housing after displacement.*

Empirically, our article is closest to the study on council estate renewal in the UK (Reades et al., 2023) but differs in two important ways. First, Reades et al. (2023) use Linked Consumer Register data to track displacement. This allows them to estimate the locations where residents move to, although individuals living in deprived circumstances are less likely to be included in the data. In contrast, we use very fine-grained administrative panel data combining socio-demographic, location, and housing variables on all individuals in the Zurich region. Second, instead of focusing solely on the renewal of public housing, we compare for-profit and non-profit redevelopment. This helps to further understand the moving patterns of residents who receive assistance in finding new housing and those who do not.

## Urban redevelopment in the Zurich region

The planning objective of reducing urban sprawl and land take to achieve environmentally sustainable urban development is widely accepted (Decoville and Feltgen, 2023). For this reason, the European Union has set the *No Net Land* target to stop land take by 2050. Today, existing spatial planning systems in European countries differ in the extent to which they align with the goal of reducing land take. Switzerland is among the countries where policies and strategies strongly support the *No Net Land* objective (D’Ascanio et al., 2024). To reduce land take, the Swiss national government revised the Spatial Planning Act in 2014, requiring municipalities to focus on the densification of urban areas (Debrunner and Hartmann, 2020). This national policy guides how urban redevelopment is implemented. Indeed, the provision of new housing is often carried out through demolition and subsequent redevelopment of the housing stock (Lutz et al., 2024). This situation raises concerns about the social sustainability of urban redevelopment, as it leads to an increasing number of terminated rental contracts (Meuth and Reutlinger, 2023).

To study how such a densification policy affects residents, we focus on the Zurich region, which includes two of the largest urban agglomerations of Switzerland, namely Zurich and Winterthur. The region comprises a total of 156 municipalities with about 1.5 million inhabitants in 2020. About 71% of all households in the Canton of Zurich live in rental housing (Kanton Zürich, 2024). This proportion lies at 90% in the City of Zurich, which is the urban center of the study region. There, 33.3% of all dwellings belong to institutional investors, such as pension funds (Stadt Zürich, 2023), and 32.1% belong to private landowners. Additionally, the City of Zurich has a high share of non-profit rental housing, usually owned by housing cooperatives (17.7% and 7.15% at the canton level). In contrast, the share of public housing is relatively low (7.0%). Hence, the Zurich region can be seen as an exemplary case of a rental housing market with a high diversity of different actors and with both a strong for-profit and non-profit rental housing sector.

Non-profit housing in the Zurich region is typically managed by housing cooperatives where residents are members of these cooperatives and thus have greater protection due to internal regulations and guidelines regarding how cooperatives implement redevelopment projects. For example, cooperatives devote time to assisting their members in finding a new apartment. This usually includes an offer for a new apartment. If tenants decline such an offer, they have to find housing on their own.

For-profit investors often redevelop so-called post-war buildings (Büttiker, 2024). In our sample, 50.9% of all demolitions were built between 1945 and 1970. Often, those typically large housing projects had not been renovated for a long time (Heye, 2007). Lower quality means lower rents, and thus developers often demolish and redevelop such buildings to generate more housing and more profit. Legally, rents in existing tenancies align with the current interest rate, however “the Swiss tenancy law system lacks nationwide reference rent regulations or capping limits, even in modernisation, renovation, or upgrades” (Debrunner et al., 2025: 13). As a result, residents often cannot afford the rent of the redeveloped buildings and are forced to relocate (Debrunner et al., 2024).

We focus on long-term residents in this article. Generally, leases are open-ended, but since the tenancy law is relatively weak when it comes to demolition or renovation, landlords can terminate rental contracts with three months’ notice (Debrunner et al., 2024). Consequently, displaced residents who lived in for-profit rental housing have to find new apartments within a relatively short time, usually without any assistance, and in a housing market where vacancy rates are low (Debrunner and Hartmann, 2020). It has become a common practice to terminate long-term contracts one or two years before the redevelopment and to rent out soon-to-be-demolished housing on a temporary basis (Debrunner and Gerber, 2021). Temporary housing is usually rented out at below market rents and to specific groups of people, such as students. We conduct additional analyses focusing on short-term residents in the Online Appendix (see section A5.7).

Taken together, the densification policy, the high share of renters, and the variety of actors involved in the provision of housing make the Zurich region an informative case to study the relocation patterns of directly displaced residents.

## Methods

In the following sections, we describe the data and the operationalization of our main variables and then present the estimation strategy.

### Data

We combine three data sources to generate detailed geo-coded panel data of all residents within the study area between 2012 and 2022. All data is provided by the Swiss Federal Statistical Office. First, we use individual income data from the Central Compensation Office (CCO) on working-age employed and self-employed residents in Switzerland. We combine this data with information on invalidity payments. Second, we combine the income data with demographic variables such as age, sex, nationality, and continent of birth from the Population and Households Statistics (STATPOP), comprising yearly administrative data starting in 2012. Third, to obtain information on residents’ housing characteristics, we use the Swiss Building Census. This data contains information on building characteristics, such as the building age, the number of apartments, and the coordinates of each building entrance, allowing us to track individuals’ addresses over time. At the dwelling level, the data specifies the square meters per flat. Additionally, starting in 2016 there is a variable indicating whether the building belongs to a for-profit or non-profit investor. To combine the different data sources, the Federal Statistical Office generates anonymized personal, building, and dwelling identification numbers. This allows us to link all data sources at the individual level and to generate geo-coded and yearly panel data with socio-demographic variables and housing information, including all approximately 1.5 million residents in the study area.

**Figure 1.**
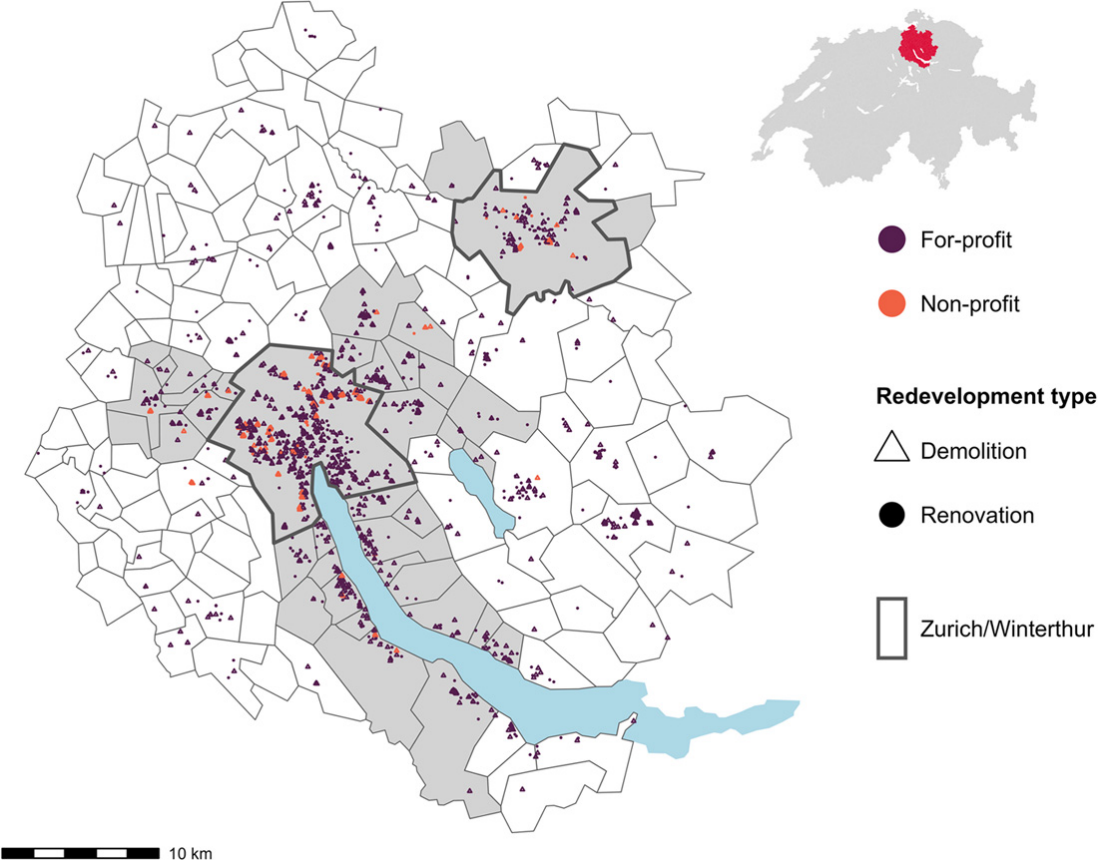
Demolished and renovated buildings in the Zurich region. This map shows all 2043 redevelopment projects in the Zurich region between 2016 and 2020. Points and triangles signify the coordinates of the building entrance of a demolished (triangle) or renovated (point) apartment building with three or more units. The tones differentiate between for-profit and non-profit investor redevelopment projects. The City of Zurich is the largest city of the region and is located by the lake. Gray areas are the core municipalities of the Zurich and Winterthur agglomeration. White areas are municipalities of the wider Zurich region. The map in the upper-right corner shows the location of the study area in Switzerland. Data: Federal Building Census and administrative borders. All sources were obtained through the Federal Statistical Office. Source: Authors’ own calculations.

Between 2016 and 2020, a total of 2043 multifamily buildings were demolished or renovated (Figure 1). We focus on multi-family buildings with three or more dwellings to ensure that owner-occupied housing redevelopments, usually consisting of single-family-homes, are excluded from the sample. Additionally, we restrict the time span to have enough pre- and post-displacement observations of each resident. A total of 346 of those buildings were non-profit and, in contrast, 1697 were for-profit redevelopments.

### Operationalization

Our independent variable is a dummy that takes the value 1 when a resident was classified as directly displaced (direct displacement = 1), namely when they had to move because of a demolition or renovation between 2016 and 2020. The comparison group (direct displacement = 0) comprises similar residents who moved for any other reason than redevelopment projects. To exclude people living in short-term rentals, we focus on residents who had been living in a building for at least three years before demolition or renovation (for an additional analysis of the group characteristics and relocation patterns of short-term residents, see Online Appendix A5.7). To determine whether displaced residents move back to a redeveloped site, we focus on where they live two years after displacement. As a robustness test, we run additional analyses based on where displaced residents live three and four years after the forced relocation (see Online Appendix A4.4 and A4.5).

We use different dependent variables. Our dependent variables on the *housing location* consist of, first, the distance between the old and new residential location. The variable is measured in log kilometers and is defined as the Euclidean distance between the coordinates of the old and the new apartment (see Online Appendix A5.8 for additional analyses focusing on the Euclidean distance to the center before and after relocation). Second, we measure local neighborhood income by calculating the median of all household incomes of residents within a 250 m radius of the new apartment, excluding income levels of directly displaced residents.

To analyze *housing characteristics*, we focus on post-war buildings and apartment size after displacement. For post-war buildings, we create a dummy variable which is equal to one when a resident moves to a building built between 1945 and 1970, and zero otherwise. As stated above, buildings constructed between 1945 and 1970 have a high likelihood of being redeveloped, and moving to such housing might result in temporary rental contracts and further displacement (we run additional analysis with a continuous building age variable and a dummy variable with 1970 as the cutoff between old and new apartments; see Online Appendix A5.5 and A5.6). Lastly, we calculate the log area consumption in square meters per person after displacement to determine whether displaced residents move to small apartments.

### Estimation strategy

Methodologically, we compare directly displaced residents to a comparison group of similar residents who moved for any other reason than housing demolition or renovation. This approach is similar to the one used by Desmond and Kimbro (2015). As displacement mostly affects low-income residents and minorities (Table 1), propensity score matching (PSM) allows us to identify for each displaced resident a resident with similar observed socioeconomic characteristics, such as sex, age, income, citizenship, residence permit, continent of birth, and a dummy whether residents were displaced from for-profit or non-profit sites (see Online Appendix A1 for more detailed information on the matching procedure). These comparison groups have similar resources for moving to a new apartment. We then compare moving patterns between these socio-demographically similar groups.

**Table 1.** Sample characteristics.

Variable	Displaced residents(*N* = 7712)	Comparison group (PSM) (*N* = 7712)	All movers(*N* = 225,229)	All residents2020(*N* = 1,277,306)
Household income(CHF per month)				
Mean (SD)	5530 (3720)	5720 (3910)	7880 (4730)	7330 (4630)
Median	4900	5110	7030	6540
Age				
Mean (SD)	44.7 (17.6)	44.9 (16.2)	39.2 (15.6)	39.6 (19.0)
Median	46.0	44.0	39.0	40.0
Sex				
Female	3699 (48.0%)	3627 (47.0%)	109,724 (48.7%)	624,316 (48.9%)
Male	4013 (52.0%)	4085 (53.0%)	115,505 (51.3%)	652,990 (51.1%)
Nationality (continent)				
Africa	136 (1.8%)	133 (1.7%)	1687 (0.7%)	13,585 (1.1%)
America (Central and South)	120 (1.6%)	111 (1.4%)	1810 (0.8%)	10,203 (0.8%)
America (North)	23 (0.3%)	11 (0.1%)	746 (0.3%)	5047 (0.4%)
Asia	320 (4.1%)	345 (4.5%)	4585 (2.0%)	32,284 (2.5%)
EU and EFTA	1640 (21.3%)	1678 (21.8%)	40,081 (17.8%)	246,159 (19.3%)
Europe (outside EU)	798 (10.3%)	778 (10.1%)	10,845 (4.8%)	72,436 (5.7%)
Swiss	4675 (60.6%)	4656 (60.4%)	165,437 (73.5%)	897,218 (70.2%)
Without nationality/no information	0 (0%)	0 (0%)	38 (0.0%)	374 (0.0%)
Continent of birth				
Africa	205 (2.7%)	210 (2.7%)	3289 (1.5%)	22,144 (1.7%)
America (Central and South)	249 (3.2%)	258 (3.3%)	5099 (2.3%)	29,435 (2.3%)
America (North)	42 (0.5%)	32 (0.4%)	1747 (0.8%)	10,921 (0.9%)
Asia	497 (6.4%)	526 (6.8%)	8648 (3.8%)	56,726 (4.4%)
EU and EFTA	1479 (19.2%)	1518 (19.7%)	36,738 (16.3%)	281,437 (18.0%)
Europe (outside EU)	833 (10.8%)	842 (10.9%)	13,330 (5.9%)	87,962 (6.9%)
Switzerland	3975 (51.5%)	3896 (50.5%)	150,730 (66.9%)	817,090 (64.0%)
Without nationality/no information	432 (5.6%)	430 (5.6%)	5648 (2.5%)	28,486 (2.2%)
Resident permit				
Asylum seeker/refugee status	63 (0.8%)	66 (0.9%)	577 (0.3%)	5164 (0.4%)
Other	6 (0.1%)	1 (0.0%)	93 (0.0%)	4462 (0.3%)
Residence permit (B)	734 (9.5%)	768 (10.0%)	17,663 (7.8%)	147,751 (11.6%)
Settlement permit (C)	2234 (29.0%)	2221 (28.8%)	41,459 (18.4%)	222,711 (17.4%)

This table shows the socio-demographic characteristics of displaced residents because of housing demolition or renovation (column 1) and the PSM comparison group (column 2). Additionally, it shows the characteristics of all residents who moved during the same time and all residents of the Zurich region (columns 3 and 4). Outliers and observations of households with more than 10 members were excluded from all samples (see Online Appendix A1 and A2).

While our research design helps to draw comparisons between residents with similar resources to move, we cannot capture the motivations for moving. Since displaced residents are forced to leave their apartments by external forces, we assume they would not move otherwise. Consequently, their motivation is very different when compared to those who move for other reasons, for example to move in with a partner or due to a job change. Our data does not allow us to understand those motives, resulting in unobserved differences between the two groups related to why residents move to a specific apartment and area. Nevertheless, given the low vacancy rates in urban centers, finding new apartments is difficult for all residents with few financial resources. Therefore, and in the absence of a natural experiment, our strategy is valuable for comparing moving patterns of residents in a similar socioeconomic situation, with the main difference between the two groups being that the treatment group is displaced and the comparison group is not.

As a first step, we compare moving patterns of those directly displaced and the comparison group descriptively. Second, we run multiple OLS-regression analyses to estimate the association between displacement and *where* and to *what* buildings displaced residents move. For the binary dependent variable whether residents move to buildings built between 1945 and 1970, we run a logistic regression (a comparison between the different regression models can be found in the Online Appendix A4.3). We include socio-demographic control variables, namely age, income, residence permit, and continent of birth, as covariates to account for the fact that, for example, individuals with lower incomes may face systematically more problems to find a new home. To control for household-specific variables, we add the floor consumption, the median income within a 250 m radius before relocation, the household size, the apartment size, and the municipality after relocation as covariates. Additionally, we include a dummy of the year of the move, to control for time-specific trends in the housing market. Standard errors are clustered at the municipality level. We then estimate the following OLS model:

(1) $$\begin{array}{c} y_{i}=\beta_{0}+\beta_{1}{Displacement}_{i}+x_{i}^{\prime }\delta +z_{i}^{\prime }\gamma \\ +\mu_{i}+\epsilon_{i} \end{array}$$

where *Y*_*i*_ stands for the different dependent variables on location and housing of individual *i* after relocation. *Displacement*_*i*_ is a dummy equal to one if individual *i* was directly displaced and zero if individual *i* belongs to the comparison group. *x*_*i*_ is a vector of the socio-demographic control variables, *δ* is a vector with the corresponding coefficients, *z*_*i*_ is a vector of the household-specific covariates with *γ* a vector with the corresponding coefficients, μ_*i*_ is the year of the move dummy, and *ϵ*_*i*_ is the error term. When holding all other variables constant, the coefficient *β*_*1*_ shows the change in the average location and housing characteristics associated with an individual being directly displaced.

Difficulties in finding a new apartment may be more pronounced in urban areas and for poor residents. As an additional analysis, we therefore rerun the regression of equation (1) described above for only the core municipalities of the agglomerations (see Figure 1) and only those residents who earn less than 60% of the median income of all movers (see Online Appendix A5.1–A5.4).

## Results

### Socio-demographic characteristics of displaced residents

Between 2016 and 2020 a total of 12,599 residents, that is, 0.8% of all residents in the Zurich region, were displaced because of housing demolitions and renovations. For the PSM, we have to drop variables with unobserved values on the matching variables. This leaves us with a total sample of 7712 observations of directly displaced residents and 7712 residents in the comparison group (see Online Appendix A1 and A2). A total of 3604 of all displaced residents, or 47%, were living in the City of Zurich. Additionally, most displaced residents were living in for-profit buildings (6569) prior to displacement, and 640 (9.7%) of them moved to non-profit housing after displacement (Table 2). In comparison, of the 1143 residents who moved from non-profit housing, 560 (49.0%) moved to non-profit housing after displacement.

**Table 2.** Housing characteristics.

Variable	Displaced for-profit(*N* = 6569)	Comparison group for-profit (*N* = 6569)	Displaced non-profit(*N* = 1143)	Comparison group non-profit (*N* = 1143)
Investor type after				
For-profit	5929 (90.3%)	6058 (92.2%)	583 (51.0%)	649 (56.8%)
Non-profit	640 (9.7%)	511 (7.8%)	560 (49.0%)	494 (43.2%)
Square meters apartment before				
Mean (SD)	77.7 (26.6)	95.9 (41.3)	67.6 (14.9)	84.5 (26.2)
Square meters apartment after				
Mean (SD)	88.2 (33.7)	101 (42.3)	83.3 (28.6)	93.4 (34.0)
Household size before				
Mean (SD)	2.91 (1.52)	3.06 (1.45)	2.69 (1.36)	3.24 (1.43)
Household size after				
Mean (SD)	2.93 (1.57)	2.86 (1.48)	2.73 (1.38)	2.99 (1.51)
Square meters per person before				
Mean (SD)	34.1 (20.0)	37.7 (21.4)	31.8 (16.4)	31.3 (16.5)
Square meters per person after				
Mean (SD)	37.5 (21.8)	42.3 (23.5)	36.8 (18.3)	37.3 (18.8)
Building construction year before				
<1945	2307 (35.1%)	1687 (25.7%)	386 (33.8%)	229 (20.0%)
1945–1970	3231 (49.2%)	1950 (29.7%)	635 (55.6%)	465 (40.7%)
>1970	1031 (15.7%)	2932 (44.6%)	122 (10.7%)	449 (39.3%)
Building construction year after				
<1945	1326 (20.2%)	1283 (19.5%)	213 (18.6%)	216 (18.9%)
1945–1970	2078 (31.6%)	1376 (20.9%)	386 (33.8%)	278 (24.3%)
>1970	3165 (48.2%)	3910 (59.5%)	544 (47.6%)	649 (56.8%)
Euclidean distance between old and new apartment (km)				
Mean (SD)	3.27 (4.64)	5.22 (6.55)	2.84 (4.27)	4.11 (5.85)
Euclidean distance to center before (km)				
Mean (SD)	7.46 (5.78)	9.07 (6.15)	4.34 (2.84)	5.69 (4.22)
Euclidean distance to center after (km)				
Mean (SD)	8.38 (5.83)	9.57 (6.25)	5.13 (3.61)	6.86 (5.16)
Median household income in CHF before (250 m radius)				
Mean (SD)	6500 (1400)	6540 (1480)	5960 (919)	5890 (1020)
Median household income in CHF after (250 m radius)				
Mean (SD)	6650 (1220)	6810 (1280)	6420 (1050)	6530 (1050)

This table shows the housing characteristics of residents displaced from for-profit housing (column 1) and from non-profit housing (column 3), with the corresponding PSM comparison group (columns 2 and 3). See Online Appendix A5.8 for additional analyses on the relocation distance. The distance to the center is the distance in km between the building coordinates and the coordinates of the closest main train station (Winterthur or Zurich main train station).

Table 1 shows the sample characteristics. Column (1) shows the characteristics of displaced residents. Column (2) shows the characteristics of the PSM group, that is, similar residents who moved for any other reason than redevelopment. Columns (3) and (4) show all movers and all residents, respectively, that is, highlighting differences between displaced residents and the general population. These findings show that displacement disproportionately affects lower-income residents and non-Swiss citizens. On average, displaced residents’ median income is 69.7% of the median income of all movers. Some 60.6% of all displaced residents hold Swiss citizenship, compared to 70.2% of all residents. The difference of 9.6 percentage points indicates that among those directly displaced there are more non-Swiss citizens. Moreover, 48.5% of all directly displaced residents were born outside of Switzerland. This share is 12.5 percentage points higher compared to the share of foreign-born residents of all residents.

### Comparison of the before and after housing situation of displaced residents

Table 2 shows an overview of the housing characteristics before and after displacement and depending on whether residents moved from for-profit or non-profit redevelopment sites. After displacement, residents from for-profit redevelopment move 12.3% further away from the urban center on average and those displaced from non-profit sites 18.2%. On average, both groups use more floor space per person after displacement (increase for-profit by 10.0%, non-profit by 15.7% square meters). Importantly, while having an increase in floor space consumption, on average displaced residents consume less floor space per person compared to all Swiss residents. The national average lies at 46 m^2^ and displaced residents use 37.5 m^2^ and 36.8 m^2^, respectively. Following displacement, residents tend to live less often in buildings built between 1945 and 1970. Together, this shows that in terms of housing characteristics, displaced residents improve their personal housing situation after displacement on average.

### Relocation patterns of displaced residents

**Figure 2.**
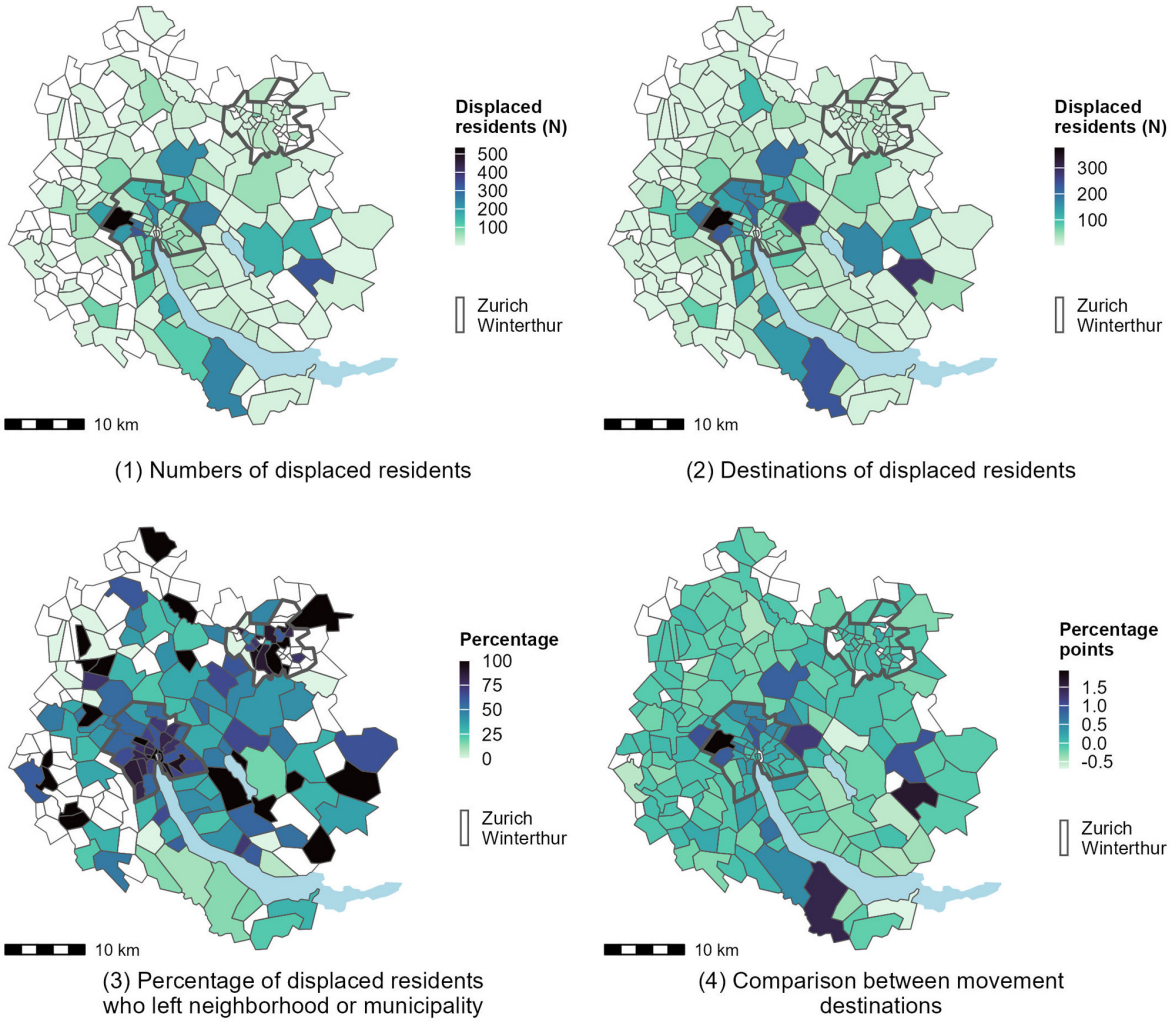
Relocation patterns of displaced residents. Map 1 shows the numbers of displaced residents per municipality and per neighborhood in the Cities of Zurich and Winterthur. Map 2 shows the numbers of where displaced residents live two years after displacement. Map 3 shows the percentage of displaced residents who had to leave their municipality or neighborhood after displacement. Map 4 shows the comparison of where displaced residents and the comparison group moved. To do so we first calculate the percentage of all displaced residents who moved to a given neighborhood/municipality and then subtract the percentage of all residents of the comparison group who moved to that same neighborhood. Data: Federal Building Census, Population and Households Statistics, and administrative borders. All sources were obtained through the Federal Statistical Office. Source: Authors’ own calculations.

Next, we focus on movement patterns (see Maps 1–4 of Figure 2). Map 1 shows the number of displaced residents per municipality. For the two largest cities, Zurich and Winterthur, we show the numbers per neighborhood. Map 2 shows where displaced residents lived two years after they were directly displaced. The maps show that locations with high numbers of displaced residents are also the ones receiving high numbers of displaced residents. This can be the case when residents move within their neighborhood or municipality or because housing is more accessible in these areas, that is, because of lower rents. Generally, displacement numbers are highest in peripheral neighborhoods of the City of Zurich and its bordering suburban municipalities. Map 3 shows the percentage of residents who left their neighborhood or municipality following displacement. This share increases in the centers of the Cities of Zurich and Winterthur, which is partly the case because of the size of the spatial units. Lastly, Map 4 compares the displaced residents’ locations following displacement to those of the comparison group. Positive values indicate that the share of displaced residents who moved to the area is larger than the share of residents of the comparison group who moved to the same area. We find positive values in the peripheral neighborhoods of the City of Zurich and its bordering municipalities where the numbers of displaced residents are comparably high. Thus, displaced residents tend to move slightly further away from the city center and mostly between the peripheral neighborhoods of the City of Zurich and its surrounding suburban municipalities.

### Regression results: Differences in post-displacement location and neighborhood characteristics of displaced residents and the PSM comparison group

**Figure 3.**
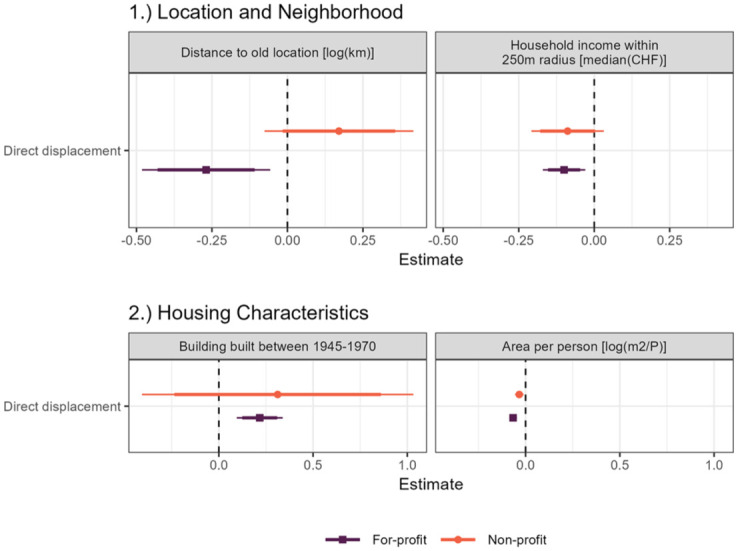
Coefficient plot regression analysis. This figure shows the regression coefficients of the regression analysis, focusing on: (1) distance between old and new apartment and neighborhood income levels within a 250 m radius after displacement (both OLS); (2) coefficients of housing characteristics (building age 1945–1970 and floor consumption per person) after displacement (logistic and OLS). The regressions include all covariates. In the area per person model, housing characteristic covariates are excluded. Standard errors are clustered at the municipality level. *N* for-profit: 13,138 and *N* non-profit: 2286. For the full regression table, see Online Appendix A3. Source: Authors’ own calculations.

Figure 3 presents the results of the regression analysis, that is, explicitly comparing displaced residents and individuals of the PSM comparison group. Panel (1) of Figure 3 shows the results of location and neighborhood characteristics following displacement. We find that displaced residents from for-profit redevelopment projects move 23.6% shorter distances than those of the comparison group. This difference is statistically significant at the 99% confidence level. Displaced residents from non-profit housing move 18.6% further from their homes than the comparison group. However, this difference is not statistically significant. The *R* ^2^ for the distance regression model is relatively low, with 0.077 for the for-profit group in contrast to 0.251 for the non-profit group. It is important to note that the location where residents move depends on the relocation process. As stated above, displaced residents of non-profit housing get an alternative apartment offered and therefore the location depends on the housing stock of the cooperative. This is not the case for those displaced from for-profit housing. Residents’ housing location after displacement likely depends on other factors that we cannot observe, such as job location, the accessibility of a neighborhood, and their friends and family network.

Residents displaced from for-profit housing moved to areas with slightly lower median household income levels within 250 m. This difference is significant at the 99.9% confidence level. In contrast, for those displaced from non-profit housing, there is no statistically significant association between displacement and neighborhood income levels.

### Regression results: Differences in post-displacement housing characteristics between displaced residents and the PSM comparison group

Panel (2) of Figure 3 shows the regression results focusing on the housing characteristics of displaced residents. Displaced residents from for-profit projects are 4% more likely to move to buildings built between 1945 and 1970 than the comparison group. The association is significant at the 99.9% confidence level. Nevertheless, the coefficient suggests a relatively weak association (see also Online Appendix A5.6).

Lastly, we focus on the floor area consumption of displaced residents and find for both for-profit and non-profit groups a significant decrease in floor area consumption compared to the respective comparison group (estimate for-profit: −0.067 equals a decrease in area per person of 6.5%; estimate non-profit: −0.034 equals a decrease in area per person of 3.3%).

In conclusion, displaced residents tend to improve their housing situation after displacement compared to their before situation (Table 2). However, when compared to similar residents, displaced residents from for-profit and non-profit redevelopment projects face worse outcomes, as they are more likely to move to smaller apartments and those displaced from for-profit sites are slightly more likely to move to buildings built between 1945 and 1970.

## Discussion and conclusion

The consequences of displacement caused by gentrification and urban redevelopment are widely studied (Banabak et al., 2024; Hochstenbach and Musterd, 2018; Reades et al., 2023; Sims and Sarmiento, 2023). However, more robust data is needed to quantify displacement and to distinguish between its various forms (Easton et al., 2020; Elliott-Cooper et al., 2020). Drawing on individual-level data from the Zurich region, we contribute to this literature by differentiating between residents displaced by for-profit and non-profit redevelopment projects.

Our findings are organized around four key points. First, direct displacement is socially stratified and disproportionately affects low-income residents. This finding aligns with previous research (Elliott-Cooper et al., 2020; Ramiller, 2022). Second, in agreement with Hypothesis 1, residents displaced from for-profit sites tend to move short distances and are more likely to relocate to lower-income neighborhoods than the PSM comparison group. This finding indicates that displaced residents prioritize proximity to their former neighborhood and social networks over neighborhood quality (Sims and Sarmiento, 2023). Third, displaced residents from for-profit housing consume less floor space after displacement and they are marginally more likely to move to post-war buildings than those of the PSM comparison group. Thus, we can support Hypothesis 2. Together, these findings highlight the need to study the neighborhood and housing characteristics after displacement in order to understand whether direct displacement increases housing inequalities (Hochstenbach and Musterd, 2018; Sims and Sarmiento, 2023). Fourth, we do not find statistically significant differences between residents displaced from non-profit sites and the PSM comparison group in terms of relocation distance, neighborhood income level, or moving into post-war buildings. However, they consumed less floor space after displacement. Thus, Hypothesis 3 can be partially accepted. We think that this is because non-profit housing organizations usually offer relocation assistance, which appears to be associated with fewer negative outcomes after direct displacement.

Beyond these findings, this study highlights the importance of identifying a relevant comparison group when examining direct displacement at an individual level. When we compare the housing situations of displaced residents before and after relocation, we find that the average floor consumption per person increases, indicating that displaced residents relocate to more favorable housing. However, this increase in floor space is likely to be associated with the demolition of post-war buildings consisting of very small rooms. Displaced residents were therefore more likely to move to newer housing after displacement, which had larger rooms on average. Nevertheless, compared to similar residents, displaced residents move to less favorable housing situations. This on first sight contradictory finding highlights the importance of selecting a comparison group when studying the impact of displacement on residents.

This article has several limitations. Our regression results on relocation distance demonstrate limited explanatory power for those moving from for-profit sites. Displaced residents face various constraints when relocating, many of which we cannot account for. Most importantly, rent levels are a key factor in determining where displaced residents can afford to move. Unfortunately, we were unable to get good enough rent data to account for this in our study. Future research should therefore account for this unobserved variable.

Our findings on the relocation of directly displaced residents are relevant for planners and policymakers in cities where housing is scarce and pressure to densify existing settlements is high. It is important to consider *who* is displaced, as redevelopment-induced displacement tends to affect vulnerable members of our societies. Understanding *where* those residents move to is crucial for a better understanding of how urban redevelopment increases housing and neighborhood inequalities. Moreover, our findings suggest that non-profit housing organizations could be supported, as they create less negative housing and neighborhood situations for displaced residents. Our findings also highlight the importance of providing support in the event of displacement. Measures could include providing alternative housing, as well as helping residents to search for new housing and ensuring they have sufficient time to do so. Overall, our findings emphasize the need for the development of policies, plans, and designs for redevelopment and densification that do not result in displacement.

## Supplemental Material

sj-docx-1-usj-10.1177_00420980251376156 – Supplemental material for Housing after redevelopment: Where and to what buildings do displaced residents move? Supplemental material, sj-docx-1-usj-10.1177_00420980251376156 for Housing after redevelopment: Where and to what buildings do displaced residents move? by Fiona Kauer, Elena Lutz and David Kaufmann in Urban Studies
