# Supplementary material for: Housing after redevelopment: Where and to what buildings do displaced residents move?
Source: Urban Stud. 2025 Oct 20;63(4):737–55. doi: 10.1177/00420980251376156 (PMC12922968; doi:10.1177/00420980251376156)
Supplement: sj-docx-1-usj-10.1177_00420980251376156 – Supplemental material for Housing after redevelopment: Where and to what buildings do displaced residents move? [file sj-docx-1-usj-10.1177_00420980251376156.docx]

**Online Appendix**

Housing after redevelopment: Where and to what buildings do displaced residents move?

**Contents**

[**A1 Propensity score matching 4**](#_Toc191564714)

[A1.1 Distribution propensity score 6](#_Toc191564715)

[A1.2 Covariate balance 7](#_Toc191564716)

[**A2 Attrition 8**](#_Toc191564717)

[**A3 Regression tables 12**](#_Toc191564718)

[A3.1 For-profit investor 12](#_Toc191564719)

[A3.2 Non-profit investor 13](#_Toc191564720)

[**A4 Robustness checks 15**](#_Toc191564721)

[A4.1 Regression models without control variables 15](#_Toc191564722)

[A4.2 Regression models with housing and neighborhood covariates 16](#_Toc191564723)

[A4.3 Comparison regression models building age DV (OLS, logit, and probit) 18](#_Toc191564724)

[A4.4 Residents housing situation three years after displacement 20](#_Toc191564725)

[A4.5 Residents housing situation four years after displacement 21](#_Toc191564726)

[**A5 Additional analyses 23**](#_Toc191564727)

[A5.1 Regression core municipalities (for-profit) 23](#_Toc191564728)

[A5.2 Regression core municipalities (non-profit) 24](#_Toc191564729)

[A5.3 Regression low-income residents (for-profit) 25](#_Toc191564730)

[A5.4 Regression low-income residents (non-profit) 26](#_Toc191564731)

[A5.5 Regression old buildings (for-profit) 28](#_Toc191564732)

[A5.6 Regression old buildings (non-profit) 29](#_Toc191564733)

[A5.7 Short-term residents 30](#_Toc191564734)

[**A5.7.1 Sample characteristics short-term residents 30**](#_Toc191564735)

[**A5.7.2 Housing characteristics short-term residents 33**](#_Toc191564736)

[**A5.7.3 Regression analyses short-term residents 37**](#_Toc191564737)

[A5.8 Distance to center 38](#_Toc191564738)

[**A5.8.1 Housing characteristics and relocation distance 39**](#_Toc191564739)

[**A5.8.2 Analyses distance to center 42**](#_Toc191564740)

[**A6 References 44**](#_Toc191564741)

# A1 Propensity score matching

Our estimation strategy relies on a selection on observables strategy, namely Propensity Score Matching (PSM). PSM is a matching technique, used to match individuals in the treatment group (i.e. displaced individuals) with similar individuals who were not displaced. This allows us to identify a comparison group for displaced residents with similar socioeconomic characteristics, such as income or citizenship (for further PSM application see: Aratani, 2011; Carlson et al., 2012; Desmond & Kimbro, 2015). We classify directly displaced residents as those who had been living in their apartments for at least three years and had to move because their apartments were demolished or renovated. All outcome variables are measured based on where those residents had been living two years after direct displacement. To create the comparison group, we identify all residents who lived in their apartments for at least three years and changed their residential location between 2016 and 2020 for any other reason than housing demolition or renovation. For consistency in the outcome variables, we include those residents’ locations and housing characteristics of where they had been living two years after the initial move.

Generally, the idea of PSM is to identify units with similar probabilities of being in the treatment group (Cunningham, 2021), in our case, being directly displaced. Specifically, we identify residents who were not displaced but have a similar probability of being in the treated group as those that were directly displaced. Based on the descriptive comparison of directly displaced residents and all individuals who had been living in the study area in 2020, we identify socio-demographic variables that correlate with being directly displaced. Note, however, that PSM only allows us to identify residents that are similar on characteristics we observe in our data, such as income, nationality, or age. There could be other unobserved factors, such as displaced individuals having a generally stronger preference for staying in their own neighborhood, which we are not able to assess.

Using this data, we calculate the propensity score including all socio-demographic covariates that knowingly cause variation in both the outcome and the selection into treatment groups (VanderWeele, 2019). Those variables are age, sex, nationality, the continent of birth, residence permit, household income, and the investor type. Including those variables, PSM estimates a maximum likelihood model of the conditional probability of treatment. Then, the predicted values are collapsed into a single scalar, namely the propensity score. This score allows to match treated with comparison units who had a similar likelihood of being assigned to the treatment. First, we do an exact match on the variable whether residents lived in a for-profit or non-profit building before displacement, to create two separate groups of (1) displaced and comparison group from for-profit sites and (2) those displaced from non-profit sites and a comparison group of residents who also lived in non-profit housing. second, we match each treated unit with the closest control unit without replacement. PSM requires common support, i.e., treated and control observations across the estimated propensity score (Cunningham, 2021). The observation of one displaced resident was removed from the sample due to this restriction. This leaves us with a sample of a total of 15,424 displaced residents and a control group of 7,712 residents. Figure A1.1 in section A1.1 shows the distribution of the propensity score and Figure A1.2 in section A1.2 shows the covariate balance of the two groups before (unadjusted) and after (adjusted) PSM.

## A1.1 Distribution propensity score

Figure A1.1 shows the distribution of the propensity scores, indicating the one treated observation (i.e. one displaced person) did not fulfil the requirement of common support and was dropped before PSM.

***Figure A1.1.*** *Distribution of propensity scores*

**
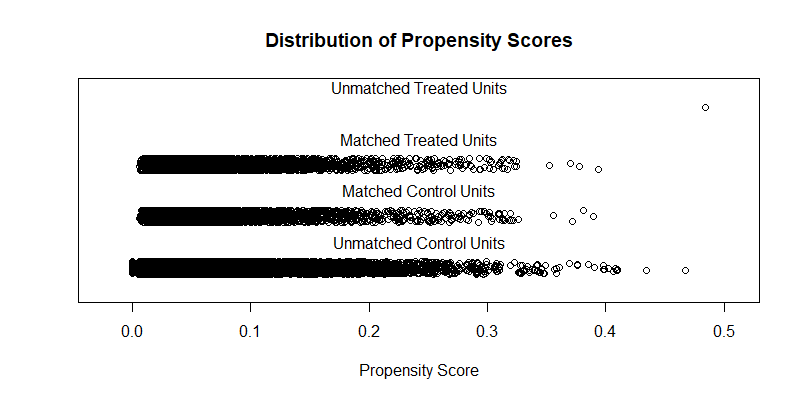
**

## A1.2 Covariate balance

Figure A1.2 shows that covariate balance after matching significantly improved on all matched variables. The variable on the project investor (variable: GEMNUTZ) was set to an exact match.

***Figure A1.2.*** *Covariate balance*


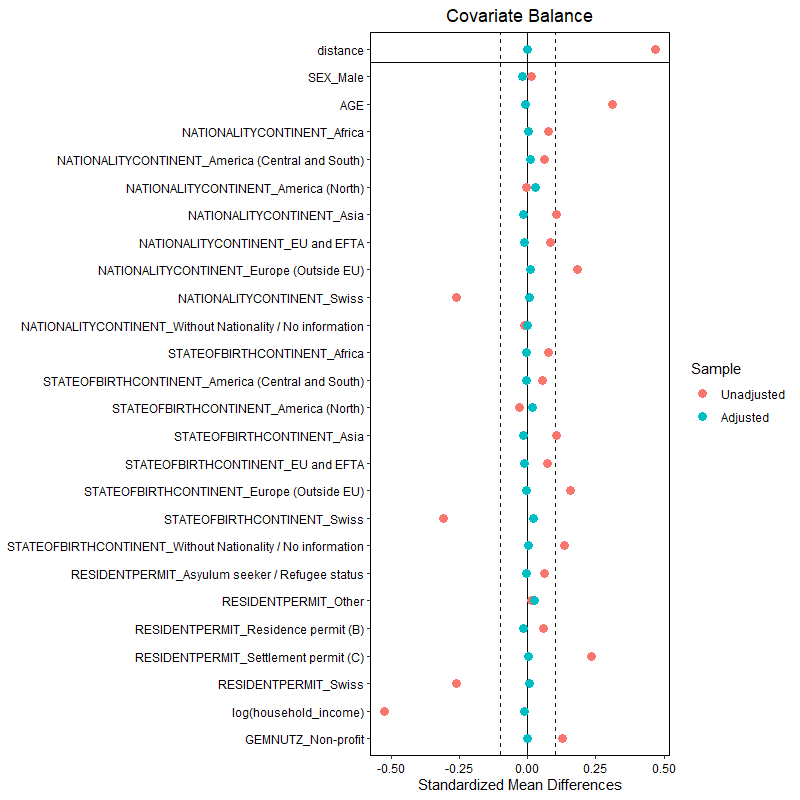


# A2 Attrition

In this section, we provide an overview of all observations that were excluded from the final sample. Generally, observations had to be removed because of four different reasons: (1) some of the residents were not present in the dataset after displacement. This can be because residents left the study region after displacement, they did not register at a new address or passed away. (2) For the PSM procedure we had to exclude all observations with NA values on either the dependent, independent or matching variables. Furthermore, (3) we exclude outliers from the data by removing the top and bottom percentile of all residents on following variables: household income, floor area consumption, and the median income of the area where they move after displacement. Additionally, (4) we excluded all observations of residents living in households together with more than 10 members to exclude institutional housing such as student or retirement homes. Table A2 shows sociodemographic and housing characteristics of all those residents that were removed from the sample due to these restrictions.

Importantly, compared to all movers a higher percentage of displaced residents left the study area after displacement, did not register at a new address, or passed away. A total of 1,643 or 13% of all displaced residents were not registered in a building located in the study area two years after displacement, compared to 3.2% of all movers (9,621). Since it is mandatory to register at a new address within two weeks after a move, it is likely that they moved to buildings outside of the study area. Hence, displaced residents were somewhat more likely to move out of the study area.

Before PSM, all observations with NA values on the dependent, independent and matching variables had to be excluded from both the group of the directly displaced residents and the potential comparison group (i.e. all residents who moved due to any other reason than building demolition or renovation). A total of 1,399 observations of the directly displaced individuals had to be removed because we did not have any information on their income levels. This is because our data has information on the income of employed and self-employed working age population (i.e. residents between 16-65). We additionally add information on invalidity payments. Nevertheless, a limitation of this data is that it does not include information on the financial situation of retired residents or unemployed residents. Therefore, those residents had to be excluded before PSM. Additionally, 153 of the displaced residents had to be removed because the floor area of the apartment before displacement was missing and for 3 residents, we could not identify the building age before displacement.

As described above, we then excluded outliers and residents living in households with more than 10 members. Table A2 shows the summary statistics on socio-demographic and housing characteristics of all those residents that either left the study area or had to be excluded from the final sample because of missing values or because they were classified as outliers. We show the values separately for those observations that were excluded from the sample of the directly displaced residents and those that had to be removed from the potential comparison group.

***Table A2.*** *Attrition*

|  | **Left study area (all movers) (N=****9621)** | **Left study area (displaced) (N=****1643)** | **NA values & outliers (all movers) (N=27802)** | **NA values & outliers (displaced) (N=1719)** |
| --- | --- | --- | --- | --- |
| **Percentage of full sample** |  |  |  |  |
| Percentage (%) | 3.2% | 13.0% | 9.2% | 13.6% |
| **Household income** |  |  |  |  |
| Mean (SD) | 9,190 (29,200) | 5,840 (18,500) | 11,400 (29,800) | 4,410 (5270) |
| Median [Min; Max] | 6,440 [30.3; 1,420,000] | 4,550 [13.7; 611,000] | 4,940 [8.08; 268,000] | 2,630 [187; 41,400] |
| Missing | 2,976 (30.9%) | 438 (26.7%) | 21,553 (77.5%) | 1,399 (81.4%) |
| **Age** |  |  |  |  |
| Mean (SD) | 58.1 (26.2) | 58.6 (22.4) | 73.0 (21.8) | 74.1 (19.4) |
| Median [Min; Max] | 53.0 [7.00; 112] | 59.0 [7.00; 112] | 79.0 [7.00; 111] | 79.0 [7.00; 104] |
| **Sex** |  |  |  |  |
| Female | 4,802 (49.9%) | 763 (46.4%) | 16,456 (59.2%) | 995 (57.9%) |
| Male | 4,819 (50.1%) | 880 (53.6%) | 11,346 (40.8%) | 724 (42.1%) |
| **Nationality (continent)** |  |  |  |  |
| Africa | 99 (1.0%) | 16 (1.0%) | 585 (2.1%) | 39 (2.3%) |
| America (Central and South) | 98 (1.0%) | 19 (1.2%) | 102 (0.4%) | 11 (0.6%) |
| America (North) | 55 (0.6%) | 12 (0.7%) | 49 (0.2%) | 0 (0%) |
| Asia | 237 (2.5%) | 60 (3.7%) | 778 (2.8%) | 57 (3.3%) |
| EU and EFTA | 2,112 (22.0%) | 406 (24.7%) | 3,278 (11.8%) | 290 (16.9%) |
| Europe (Outside EU) | 384 (4.0%) | 128 (7.8%) | 752 (2.7%) | 69 (4.0%) |
| Swiss | 6,633 (68.9%) | 1,002 (61.0%) | 22,246 (80.0%) | 1,246 (72.5%) |
| Without Nationality / No information | 3 (0.0%) | 0 (0%) | 12 (0.0%) | 7 (0.4%) |
| **Continent of birth** |  |  |  |  |
| Africa | 164 (1.7%) | 37 (2.3%) | 696 (2.5%) | 51 (3.0%) |
| America (Central and South) | 229 (2.4%) | 40 (2.4%) | 332 (1.2%) | 27 (1.6%) |
| America (North) | 95 (1.0%) | 17 (1.0%) | 140 (0.5%) | 3 (0.2%) |
| Asia | 415 (4.3%) | 98 (6.0%) | 1,093 (3.9%) | 75 (4.4%) |
| EU and EFTA | 2,406 (25.0%) | 454 (27.6%) | 5,473 (19.7%) | 440 (25.6%) |
| Europe (Outside EU) | 445 (4.6%) | 116 (7.1%) | 713 (2.6%) | 57 (3.3%) |
| Swiss | 5,679 (59.0%) | 799 (48.6%) | 18,728 (67.4%) | 990 (57.6%) |
| Without Nationality / No information | 188 (2.0%) | 82 (5.0%) | 627 (2.3%) | 76 (4.4%) |
| **Resident permit** |  |  |  |  |
| Asylum seeker / Refugee status | 42 (0.4%) | 14 (0.9%) | 714 (2.6%) | 80 (4.7%) |
| Other | 23 (0.2%) | 7 (0.4%) | 86 (0.3%) | 0 (0%) |
| Residence permit (B) | 1,110 (11.5%) | 204 (12.4%) | 1,544 (5.6%) | 49 (2.9%) |
| Settlement permit (C) | 1,813 (18.8%) | 416 (25.3%) | 3,212 (11.6%) | 344 (20.0%) |
| Swiss | 6,633 (68.9%) | 1,002 (61.0%) | 22,246 (80.0%) | 1,246 (72.5%) |
| **Investor type before** |  |  |  |  |
| For-profit | 8,540 (88.8%) | 1,421 (86.5%) | 23,827 (85.7%) | 1,428 (83.1%) |
| Non-profit | 1,079 (11.2%) | 222 (13.5%) | 3,960 (14.2%) | 291 (16.9%) |
| Missing | 2 (0.0%) | 0 (0%) | 15 (0.1%) | 0 (0%) |
| **Square meters apartment before** |  |  |  |  |
| Mean (SD) | 96.7 (47.6) | 72.0 (29.6) | 96.0 (47.2) | 70.6 (26.8) |
| Median [Min; Max] | 88.0 [12.0; 650] | 70.0 [10.0; 350] | 85.0 [10.0; 580] | 68.0 [12.0; 221] |
| Missing | 330 (3.4%) | 49 (3.0%) | 3520 (12.7%) | 153 (8.9%) |
| **Household size before** |  |  |  |  |
| Mean (SD) | 8.18 (104) | 2.62 (4.31) | 17.1 (161) | 3.32 (9.76) |
| Median [Min; Max] | 2.00 [1.00; 3180] | 2.00 [1.00; 55.0] | 2.00 [1.00; 3,180] | 1.00 [1.00; 86.0] |
| **Square meters per person**  **before** |  |  |  |  |
| Mean (SD) | 51.0 (31.3) | 43.8 (26.5) | 65.1 (37.6) | 51.8 (24.0) |
| Median [Min; Max] | 43.0 [2.33; 380] | 36.0 [5.00; 220] | 58.5 [3.33; 480] | 48.8 [4.17; 183] |
| Missing | 330 (3.4%) | 49 (3.0%) | 3,520 (12.7%) | 153 (8.9%) |
| **Building construction year before** |  |  |  |  |
| <1945 | 2222 (23.1%) | 679 (41.3%) | 7362 (26.5%) | 522 (30.4%) |
| 1945-1970 | 2920 (30.4%) | 748 (45.5%) | 8831 (31.8%) | 891 (51.8%) |
| >1970 | 4479 (46.6%) | 216 (13.1%) | 11609 (41.8%) | 306 (17.8%) |
| **Median household income in CHF (250m radius)** |  |  |  |  |
| Mean (SD) | NA | NA | 6,760 (2,020) | 6,600 (1,470) |
| Median [Min, Max] | NA | NA | 6,490 [30.5; 176,000] | 6,390 [1,340; 23,400] |
| Missing | 9,621 (100%) | 1,643 (100%) | 84 (0.3%) | 0 (0%) |
| **Note:** Column 1 and 2 shows summary statistics for all residents who moved to a municipality outside the study region. Column 3 and 4 combines observations with NA values on either the dependent, independent or matching variable, no restrictions on household size, and all observations that were classified as outliers, i.e., above 99 percentile or below 1 percentile on the following variables household income, floor area consumption, and the median income of the area where residents moved (after displacement). | | | | |

# A3 Regression tables

In this section, we first show the full regression table of the main analysis including all covariates. We show the results separately for those displaced from for-profit sites and those displaced from non-profit sites.

## A3.1 For-profit investor

| **Table A3.1.** For-profit Redevelopment: Displacement due to housing demolition or redevelopment | | | | |
| --- | --- | --- | --- | --- |
|  | | | | |
|  | *Dependent variable:* | | | |
|  |  | | | |
|  | Distance to old location [log(km)] | Building built between 1945-1970 | Area per person [log(m2/P)] | Household income within 250m radius [median(CHF)] |
|  | OLS | *Logistic* | *OLS* | *OLS* |
|  | (1) | (2) | (3) | (4) |
|  | | | | |
| **Direct displacement** | **-0.269^**^ (0.081)** | **0.217^***^ (0.047)** | **-0.067^***^ (0.008)** | **-0.100^***^ (0.027)** |
| Log household income | -0.0002 (0.050) | -0.183^***^ (0.038) | 0.080^***^ (0.006) | 0.103^***^ (0.025) |
| Age | -0.006^***^ (0.001) | -0.0001 (0.002) | 0.001^**^ (0.0004) | 0.00004 (0.001) |
| Resident permit [other] | 0.154 (0.777) | -1.186 (1.479) | 0.049 (0.128) | -0.438 (0.316) |
| Resident permit [B] | -0.005 (0.194) | 0.305 (0.396) | -0.065^+^ (0.035) | 0.159^*^ (0.080) |
| Resident permit [C] | -0.177 (0.191) | 0.274 (0.390) | -0.051 (0.034) | 0.103 (0.089) |
| Resident permit [Swiss] | -0.150 (0.185) | 0.242 (0.369) | 0.017 (0.032) | 0.093 (0.079) |
| State of birth [America (Central and South)] | 0.272^*^ (0.126) | 0.130 (0.171) | -0.022 (0.017) | 0.132^**^ (0.049) |
| State of birth [America (North)] | -0.275 (0.231) | 0.479^+^ (0.272) | 0.099^**^ (0.038) | 0.285^*^ (0.140) |
| State of birth [Asia] | 0.211^*^ (0.102) | -0.048 (0.178) | 0.013 (0.015) | 0.138^*^ (0.065) |
| State of birth [EU and EFTA] | 0.048 (0.110) | -0.146 (0.161) | 0.047^**^ (0.016) | 0.170^*^ (0.066) |
| State of birth [Europe (Outside EU)] | -0.162 (0.128) | -0.134 (0.166) | -0.018 (0.015) | 0.042 (0.071) |
| State of birth [Switzerland] | -0.120 (0.095) | -0.273 (0.193) | 0.062^***^ (0.012) | 0.153^*^ (0.059) |
| State of birth [Without Nationality / No information] | 0.139 (0.249) | -0.167 (0.139) | 0.001 (0.015) | 0.016 (0.054) |
| Household members | -0.103^***^ (0.018) | 0.016 (0.033) | -0.191^***^ (0.005) | 0.009 (0.018) |
| Log area per person before | 0.146^*^ (0.068) | -0.297^***^ (0.056) | 0.160^***^ (0.010) | 0.180^***^ (0.022) |
| Area | 0.002^**^ (0.001) | -0.017^***^ (0.002) |  | 0.005^***^ (0.0005) |
| Building built between 1945-1970 (before) | 0.022 (0.072) | 0.560^***^ (0.103) | -0.015 (0.011) | -0.041^+^ (0.024) |
| Median income 250m radius (before) | 0.045^+^ (0.026) | -0.002 (0.024) | 0.018^***^ (0.003) | 0.165^***^ (0.047) |
|  | | | | |
| Observations | 13,138 | 13,138 | 13,138 | 13,138 |
| R^2^ | 0.077 |  | 0.600 | 0.435 |
| Adjusted R^2^ | 0.064 |  | 0.594 | 0.427 |
| Log Likelihood |  | -6,539.535 |  |  |
| Akaike Inf. Crit. |  | 13,435.070 |  |  |
|  | | | | |
| *Note:* | Regression with all individual-level and housing unit covariates on housing location and characteristics after displacement. Sample includes all residents displaced from for-profit redevelopment projects in the Zurich region. Standard errors are clustered at the municipality level. ***p < 0.001, **p < 0.01, *p < 0.05, +p < 0.1 | | | |

## A3.2 Non-profit investor

| ***Table A3.2.*** *Non-profit Redevelopment: Displacement due to housing demolition or redevelopment* | | | | |
| --- | --- | --- | --- | --- |
|  | | | | |
|  | *Dependent variable:* | | | |
|  |  | | | |
|  | Distance to old location [log(km)] | Building built between 1945-1970 | Area per person [log(m2/P)] | Household income within 250m radius [median(CHF)] |
|  | *OLS* | *Logistic* | *OLS* | *OLS* |
|  | (1) | (2) | (3) | (4) |
|  | | | | |
| **Direct displacement** | **0.171^+^ (0.094)** | **0.312 (0.279)** | **-0.034^***^ (0.009)** | **-0.088^+^ (0.046)** |
| Log household income | 0.053 (0.061) | -0.193^**^ (0.069) | 0.049^***^ (0.007) | 0.011 (0.034) |
| Age | -0.006^**^ (0.002) | -0.003 (0.005) | 0.001^***^ (0.0003) | -0.003^*^ (0.001) |
| Resident permit [B] | 2.107 (1.576) | 0.312 (1.067) | -0.054 (0.047) | 0.725 (0.475) |
| Resident permit [C] | 1.642 (1.540) | 0.489 (1.133) | 0.019 (0.038) | 0.650 (0.467) |
| Resident permit [Swiss] | 1.607 (1.569) | 0.219 (1.102) | 0.020 (0.038) | 0.679 (0.468) |
| State of birth [America (Central and South)] | 0.333 (0.204) | 0.253 (0.255) | 0.069^+^ (0.038) | -0.223^**^ (0.084) |
| State of birth [America (North)] | -0.598^+^ (0.352) | 0.962 (0.745) | 0.051 (0.038) | 0.156^*^ (0.077) |
| State of birth [Asia] | 0.154 (0.186) | 0.213 (0.275) | 0.072^*^ (0.030) | -0.021 (0.110) |
| State of birth [EU and EFTA] | -0.024 (0.151) | -0.149 (0.220) | 0.039 (0.029) | -0.113 (0.092) |
| State of birth [Europe (Outside EU)] | 0.196 (0.252) | 0.064 (0.469) | 0.025 (0.045) | -0.077 (0.086) |
| State of birth [Switzerland] | 0.102 (0.152) | 0.116 (0.191) | 0.104^***^ (0.025) | -0.132 (0.101) |
| State of birth [Without Nationality / No information] | 0.179 (0.133) | 0.159 (0.249) | 0.046 (0.033) | -0.105 (0.111) |
| Household members | -0.139^*^ (0.066) | 0.196^*^ (0.091) | -0.196^***^ (0.012) | -0.032^+^ (0.019) |
| Log area per person before | 0.187^+^ (0.100) | -0.164 (0.192) | 0.128^***^ (0.021) | 0.194^***^ (0.042) |
| Area | 0.006^***^ (0.002) | -0.037^***^ (0.008) |  | 0.005^***^ (0.001) |
| Building built between 1945-1970 (before) | 0.134 (0.099) | 0.945^**^ (0.322) | -0.020^*^ (0.009) | -0.064 (0.059) |
| Median income 250m radius (before) | -0.272^***^ (0.041) | -0.072 (0.055) | 0.014^**^ (0.005) | 0.256^***^ (0.050) |
|  | | | | |
| Observations | 2,286 | 2,286 | 2,286 | 2,286 |
| R^2^ | 0.251 |  | 0.634 | 0.280 |
| Adjusted R^2^ | 0.203 |  | 0.611 | 0.234 |
| Log Likelihood |  | -1,062.834 |  |  |
| Akaike Inf. Crit. |  | 2401.7 |  |  |
|  | | | | |
| *Note:* | Regression with all individual-level and housing unit covariates on housing location and characteristics after displacement. Sample includes all residents displaced from non-profit redevelopment projects in the Zurich region. Standard errors are clustered at the municipality level. ***p < 0.001, **p < 0.01, *p < 0.05, +p < 0.1 | | | |

# A4 Robustness checks

## A4.1 Regression models without control variables

| ***Table A4.1.1.*** *For-profit redevelopment: Displacement due to housing demolition or redevelopment* | | | | |
| --- | --- | --- | --- | --- |
|  | | | | |
|  | *Dependent variable:* | | | |
|  |  | | | |
|  | Distance to old location [log(km)] | Building built between 1945-1970 | Area per person [log(m2/P)] | Household income within 250m radius [median(CHF)] |
|  | *OLS* | *Logistic* | *OLS* | *OLS* |
|  | (1) | (2) | (3) | (4) |
|  | | | | |
| **Direct displacement** | **-0.427^***^ (0.031)** | **0.557^***^ (0.040)** | **-0.129^***^ (0.009)** | **-0.157^***^ (0.022)** |
| Housing and neighborhood covariates | No | No | No | No |
| Individual-level covariates | No | No | No | No |
|  | | | | |
| Observations | 13,138 | 13,138 | 13,138 | 13,138 |
| R^2^ | 0.014 |  | 0.016 | 0.004 |
| Adjusted R^2^ | 0.014 |  | 0.016 | 0.004 |
| Log Likelihood |  | -7,471.103 |  |  |
| Akaike Inf. Crit. |  | 14,946.210 |  |  |
|  | | | | |
| *Note:* | Regressions without covariates on housing location and characteristics after displacement. The sample includes all residents displaced from for-profit redevelopment projects in the Zurich region. Standard errors in brackets. ***p < 0.001, **p < 0.01, *p < 0.05, +p < 0.1 | | | |

| ***Table A4.1.2.*** *Non-profit redevelopment: Displacement due to housing demolition or redevelopment* | | | | |
| --- | --- | --- | --- | --- |
|  | | | | |
|  | *Dependent variable:* | | | |
|  |  | | | |
|  | Distance to old location [log(km)] | Building built between 1945-1970 | Area per person [log(m2/P)] | Household income within 250m radius [median(CHF)] |
|  | *OLS* | *Logistic* | *OLS* | *OLS* |
|  | (1) | (2) | (3) | (4) |
|  | | | | |
| **Direct displacement** | **-0.162^*^ (0.080)** | **0.462^***^ (0.093)** | **-0.018 (0.019)** | **-0.108^*^ (0.044)** |
| Housing and neighborhood covariates | No | No | No | No |
| Individual-level covariates | No | No | No | No |
|  | | | | |
| Observations | 2,286 | 2,286 | 2,286 | 2,286 |
| R^2^ | 0.002 |  | 0.0004 | 0.003 |
| Adjusted R^2^ | 0.001 |  | -0.00004 | 0.002 |
| Log Likelihood |  | -1,365.046 |  |  |
| Akaike Inf. Crit. |  | 2,734.092 |  |  |
|  | | | | |
| *Note:* | Regressions without covariates on housing location and characteristics after displacement. The sample includes all residents displaced from non-profit redevelopment projects in the Zurich region. Standard errors in brackets. ***p < 0.001, **p < 0.01, *p < 0.05, +p < 0.1 | | | |

## A4.2 Regression models with housing and neighborhood covariates

| ***Table A4.2.1.*** *For-profit redevelopment: Displacement due to housing demolition or redevelopment* | | | | |
| --- | --- | --- | --- | --- |
|  | | | | |
|  | *Dependent variable:* | | | |
|  |  | | | |
|  | Distance to old location [log(km)] | Building built between 1945-1970 | Area per person [log(m2/P)] | Household income within 250m radius [median(CHF)] |
|  | *OLS* | *Logistic* | *OLS* | *OLS* |
|  | (1) | (2) | (3) | (4) |
|  | | | | |
| **Direct displacement** | **-0.288^**^ (0.087)** | **0.190^***^ (0.047)** | **-0.057^***^ (0.010)** | **-0.092^**^ (0.028)** |
| Housing and neighborhood covariates | Yes | Yes | Yes | Yes |
| Individual-level covariates | No | No | No | No |
|  | | | | |
| Observations | 13,138 | 13,138 | 13,138 | 13,138 |
| R^2^ | 0.069 |  | 0.574 | 0.428 |
| Adjusted R^2^ | 0.058 |  | 0.569 | 0.421 |
| Log Likelihood |  | -6,578.593 |  |  |
| Akaike Inf. Crit. |  | 13,487.190 |  |  |
|  | | | | |
| *Note:* | Regressions with housing unit and neighborhood covariates on housing location and characteristics after displacement. The sample includes all residents displaced from for-profit redevelopment projects in the Zurich region. Standard errors are clustered at the municipality level. ***p < 0.001, **p < 0.01, *p < 0.05, +p < 0.1 | | | |

| ***Table A4.2.2.*** *Non-profit redevelopment: Displacement due to housing demolition or redevelopment* | | | | |
| --- | --- | --- | --- | --- |
|  | | | | |
|  | *Dependent variable:* | | | |
|  |  | | | |
|  | Distance to old location [log(km)] | Building built between 1945-1970 | Area per person [log(m2/P)] | Household income within 250m radius [median(CHF)] |
|  | *OLS* | *Logistic* | *OLS* | *OLS* |
|  | (1) | (2) | (3) | (4) |
|  | | | | |
| **Direct displacement** | **0.174^+^ (0.096)** | **0.277 (0.285)** | **-0.032^**^ (0.010)** | **-0.090^+^ (0.047)** |
| Housing and neighborhood covariates | Yes | Yes | Yes | Yes |
| Individual-level covariates | No | No | No | No |
|  | | | | |
| Observations | 2,286 | 2,286 | 2,286 | 2,286 |
| R^2^ | 0.241 |  | 0.620 | 0.276 |
| Adjusted R^2^ | 0.197 |  | 0.598 | 0.234 |
| Log Likelihood |  | -1,070.114 |  |  |
| Akaike Inf. Crit. |  | 2,392.228 |  |  |
|  | | | | |
| *Note:* | Regressions with housing unit and neighborhood covariates on housing location and characteristics after displacement. The sample includes all residents displaced from non-profit redevelopment projects in the Zurich region. Standard errors are clustered at the municipality level. ***p < 0.001, **p < 0.01, *p < 0.05, +p < 0.1 | | | |

## A4.3 Comparison regression models building age DV (OLS, logit, and probit)

In this section we first show the regression table with three different model specifications (OLS, logit and probit). Second, we show the comparison of the average marginal effects. The for-profit regression results are presented before the non-profit results. The comparison of the average marginal effects shows that the results remain very similar across the different model specifications.

| ***Table A4.3.1.*** *For-profit redevelopment: Displacement due to housing demolition or redevelopment* | | | |
| --- | --- | --- | --- |
|  | | | |
|  | *Dependent variable: Building built between 1945-1970* | | |
|  |  | | |
|  | *OLS* | *Probit* | *Logistic* |
|  | (1) | (2) | (3) |
|  | | | |
| **Direct displacement** | **0.039^***^ (0.008)** | **0.130^***^ (0.028)** | **0.217^***^ (0.047)** |
| Housing and neighborhood covariates | Yes | Yes | Yes |
| Individual-level covariates | Yes | Yes | Yes |
|  | | | |
| Observations | 13,138 | 13,138 | 13,138 |
| R^2^ | 0.132 |  |  |
| Adjusted R^2^ | 0.120 |  |  |
| Log Likelihood |  | -6,557.916 | -6,539.535 |
| Akaike Inf. Crit. |  | 13,471.830 | 13,435.070 |
|  | | | |
| *Note:* | Model comparison of regression analysis on the association between direct displacement and moving to a building built between 1945-1970. The sample includes all residents displaced from for-profit redevelopment projects in the Zurich region. Standard errors are clustered at the municipality level. ***p < 0.001, **p < 0.01, *p < 0.05, +p < 0.1 | | |

| ***Table A4.3.2.*** *Average marginal effect* | | | |
| --- | --- | --- | --- |
|  | *OLS* | *Probit* | *Logistic* |
|  | (1) | (2) | (3) |
|  | | | |
| **Direct displacement** | **0.04 ***** | **0.04 ***** | **0.04 ***** |
| Housing and neighborhood covariates | Yes | Yes | Yes |
| Individual-level covariates | Yes | Yes | Yes |
|  | | | |
|  | | | |
| *Note:* | | Comparison of the average marginal effect | |

| **Table A4.3.3**. Non-profit redevelopment: Displacement due to housing demolition or redevelopment | | | |
| --- | --- | --- | --- |
|  | | | |
|  | *Dependent variable: Building built between 1945-1970* | | |
|  |  | | |
|  | *OLS* | *Probit* | *Logistic* |
|  | (1) | (2) | (3) |
|  | | | |
| **Direct displacement** | **0.043 (0.050)** | **0.168 (0.168)** | **0.312 (0.279)** |
| Housing and neighborhood covariates | Yes | Yes | Yes |
| Individual-level covariates | Yes | Yes | Yes |
|  | | | |
| Observations | 2,286 | 2,286 | 2,286 |
| R^2^ | 0.223 |  |  |
| Adjusted R^2^ | 0.174 |  |  |
| Log Likelihood |  | -1,068.376 | -1,062.834 |
| Akaike Inf. Crit. |  | 2,412.753 | 2,401.667 |
|  | | | |
| *Note:* | Model comparison of regression analysis on the association between direct displacement and moving to a building built between 1945-1970. The sample includes all residents displaced from non-profit redevelopment projects in the Zurich region. Standard errors are clustered at the municipality level. ***p < 0.001, **p < 0.01, *p < 0.05, +p < 0.1 | | |

| ***Table A4.3.4****. Average marginal effect* | | | |
| --- | --- | --- | --- |
|  | *OLS* | *Probit* | *Logistic* |
|  | (1) | (2) | (3) |
|  | | | |
| **Direct displacement** | **0.04** | **0.04** | **0.05** |
| Housing and neighborhood covariates | Yes | Yes | Yes |
| Individual-level covariates | Yes | Yes | Yes |
|  | | | |
|  | | | |
| *Note:* | | Comparison of the average marginal effect | |

## A4.4 Residents housing situation three years after displacement

| ***Table A4.4.1****. For-profit redevelopment: Displacement due to housing demolition or redevelopment* | | | | |
| --- | --- | --- | --- | --- |
|  | | | | |
|  | *Dependent variable:* | | | |
|  |  | | | |
|  | Distance to old location [log(km)] | Building built between 1945-1970 | Area per person [log(m2/P)] | Household income within 250m radius [median(CHF)] |
|  | *OLS* | *Logistic* | *OLS* | *OLS* |
|  | (1) | (2) | (3) | (4) |
|  | | | | |
| **Direct displacement** | **-0.260^**^ (0.086)** | **0.168^*^ (0.067)** | **-0.055^***^ (0.009)** | **-0.126^***^ (0.034)** |
| Housing and neighborhood covariates | Yes | Yes | Yes | Yes |
| Individual-level covariates | Yes | Yes | Yes | Yes |
|  | | | | |
| Observations | 10,208 | 10,208 | 10,208 | 10,208 |
| R^2^ | 0.074 |  | 0.607 | 0.445 |
| Adjusted R^2^ | 0.057 |  | 0.600 | 0.435 |
| Log Likelihood |  | -5,026.852 |  |  |
| Akaike Inf. Crit. |  | 10,405.700 |  |  |
|  | | | | |
| *Note:* | Regression with all individual-level and housing unit covariates on housing location and characteristics after displacement. Sample includes all residents displaced from for-profit redevelopment projects in the Zurich region. Standard errors are clustered at the municipality level. ***p < 0.001, **p < 0.01, *p < 0.05, +p < 0.1 | | | |

| ***Table A4.4.2.*** *Non-profit redevelopment: Displacement due to housing demolition or redevelopment* | | | | |
| --- | --- | --- | --- | --- |
|  | | | | |
|  | *Dependent variable:* | | | |
|  |  | | | |
|  | Distance to old location [log(km)] | Building built between 1945-1970 | Area per person [log(m2/P)] | Household income within 250m radius [median(CHF)] |
|  | *OLS* | *Logistic* | *OLS* | *OLS* |
|  | (1) | (2) | (3) | (4) |
|  | | | | |
| **Direct displacement** | **0.313^*^ (0.128)** | **0.035 (0.309)** | **-0.044^**^ (0.014)** | **-0.107^**^ (0.036)** |
| Housing and neighborhood covariates | Yes | Yes | Yes | Yes |
| Individual-level covariates | Yes | Yes | Yes | Yes |
|  | | | | |
| Observations | 1,771 | 1,771 | 1,771 | 1,771 |
| R^2^ | 0.275 |  | 0.629 | 0.299 |
| Adjusted R^2^ | 0.220 |  | 0.601 | 0.246 |
| Log Likelihood |  | -799.784 |  |  |
| Akaike Inf. Crit. |  | 1,851.567 |  |  |
|  | | | | |
| *Note:* | Regression with all individual-level and housing unit covariates on housing location and characteristics after displacement. Sample includes all residents displaced from non-profit redevelopment projects in the Zurich region. Standard errors are clustered at the municipality level. ***p < 0.001, **p < 0.01, *p < 0.05, +p < 0.1 | | | |

## A4.5 Residents housing situation four years after displacement

| ***Table A4.5.1.*** *For-profit redevelopment: Displacement due to housing demolition or redevelopment* | | | | |
| --- | --- | --- | --- | --- |
|  | | | | |
|  | *Dependent variable:* | | | |
|  |  | | | |
|  | Distance to old location [log(km)] | Building built between 1945-1970 | Area per person [log(m2/P)] | Household income within 250m radius [median(CHF)] |
|  | *OLS* | *Logistic* | *OLS* | *OLS* |
|  | (1) | (2) | (3) | (4) |
|  | | | | |
| **Direct displacement** | **-0.215^*^ (0.106)** | **0.215^*^ (0.096)** | **-0.059^***^ (0.010)** | **-0.124^**^ (0.038)** |
| Housing and neighborhood covariates | Yes | Yes | Yes | Yes |
| Individual-level covariates | Yes | Yes | Yes | Yes |
|  | | | | |
| Observations | 7,072 | 7,072 | 7,072 | 7,072 |
| R^2^ | 0.093 |  | 0.600 | 0.457 |
| Adjusted R^2^ | 0.070 |  | 0.590 | 0.444 |
| Log Likelihood |  | -3,415.173 |  |  |
| Akaike Inf. Crit. |  | 7,176.345 |  |  |
|  | | | | |
| *Note:* | Regression with all individual-level and housing unit covariates on housing location and characteristics after displacement. Sample includes all residents displaced from for-profit redevelopment projects in the Zurich region. Standard errors are clustered at the municipality level. ***p < 0.001, **p < 0.01, *p < 0.05, +p < 0.1 | | | |

| ***Table A4.5.2.*** *Non-profit redevelopment: Displacement due to housing demolition or redevelopment* | | | | |
| --- | --- | --- | --- | --- |
|  | | | | |
|  | *Dependent variable:* | | | |
|  |  | | | |
|  | Distance to old location [log(km)] | Building built between 1945-1970 | Area per person [log(m2/P)] | Household income within 250m radius [median(CHF)] |
|  | *OLS* | *Logistic* | *OLS* | *OLS* |
|  | (1) | (2) | (3) | (4) |
|  | | | | |
| **Direct displacement** | **0.208 (0.223)** | **0.060 (0.305)** | **-0.058^***^ (0.013)** | **-0.075 (0.057)** |
| Housing and neighborhood covariates | Yes | Yes | Yes | Yes |
| Individual-level covariates | Yes | Yes | Yes | Yes |
|  | | | | |
| Observations | 1,349 | 1,349 | 1,349 | 1,349 |
| R^2^ | 0.281 |  | 0.660 | 0.311 |
| Adjusted R^2^ | 0.213 |  | 0.628 | 0.246 |
| Log Likelihood |  | -579.913 |  |  |
| Akaike Inf. Crit. |  | 1,393.827 |  |  |
|  | | | | |
| *Note:* | Regression with all individual-level and housing unit covariates on housing location and characteristics after displacement. Sample includes all residents displaced from non-profit redevelopment projects in the Zurich region. Standard errors are clustered at the municipality level. ***p < 0.001, **p < 0.01, *p < 0.05, +p < 0.1 | | | |

# A5 Additional analyses

This Section presents additional analyses. First, we run the analysis for residents who were displaced from the core municipalities of the urban agglomerations in the Zurich region (sections A5.1 and A5.2). The core municipalities are shown in Figure 1 in the main text. We run these analyses because housing is generally more expensive in the core municipalities, which means that displaced residents in the core municipalities may find it more difficult to remain close to their old homes. Next, we present the regression tables that include only those residents whose monthly income is less than 60% of the median income of all residents who moved (sections A5.3 and A5.4). This particularly vulnerable group may have different outcomes than the overall sample. In section A5.5 and A5.6 we test alternative specifications for the building age variable. In section A5.7 we focus on short-term residents. We first discuss the differences between short-term and long-term residents and then present the summary statistics and regression analysis for all short-term residents who had to move because of housing demolitions or renovations. Finally, section A5.8 presents the analysis focusing on the distance to the center before and after displacement. All analyses include the following covariates: household income, age, residence permit, continent of birth, household size, floor area, and area consumption per person, municipality, and the year of the move.

## A5.1 Regression core municipalities (for-profit)

Our results remain similar when we restrict the sample to the core municipalities of the largest urban agglomerations in the Zurich region. In particular, most of the directly displaced residents (82%) lived in these core municipalities before being displaced. This suggests that direct displacement due to housing demolition or renovation in the Zurich region is an urban phenomenon, occurring most frequently in dense and urban areas.

| ***Table A5.1****. For-profit redevelopment: Displacement due to housing demolition or redevelopment* | | | | |
| --- | --- | --- | --- | --- |
|  | | | | |
|  | *Dependent variable:* | | | |
|  |  | | | |
|  | Distance to old location [log(km)] | Building built between 1945-1970 | Area per person [log(m2/P)] | Household income within 250m radius [median(CHF)] |
|  | *OLS* | *Logistic* | *OLS* | *OLS* |
|  | (1) | (2) | (3) | (4) |
|  | | | | |
| **Direct displacement** | **-0.210^**^ (0.072)** | **0.233*** (0.058)** | **-0.070*** (0.010)** | **-0.121*** (0.030)** |
| Housing and neighborhood covariates | Yes | Yes | Yes | Yes |
| Individual-level covariates | Yes | Yes | Yes | Yes |
|  | | | | |
| Observations | 9,283 | 9,283 | 9,283 | 9,283 |
| R^2^ | 0.204 |  | 0.599 | 0.413 |
| Adjusted R^2^ | 0.189 |  | 0.591 | 0.402 |
| Log Likelihood |  | -4,839.486 |  |  |
| Akaike Inf. Crit. |  | 10,018.970 |  |  |
|  | | | | |
| *Note:* | Regression with all individual-level and housing unit covariates on housing location and characteristics after displacement. Sample includes all residents displaced from for-profit redevelopment projects in core municipalities of the Zurich region. Standard errors are clustered at the municipality level. ***p < 0.001, **p < 0.01, *p < 0.05, +p < 0.1 | | | |

## A5.2 Regression core municipalities (non-profit)

Most non-profit projects in the Zurich region are located within the largest cities of Zurich and Winterthur. Therefore, most displaced residents from such sites were living in one of the core municipalities of our study region before displacement. As expected, the results for those displaced from non-profit sites remain very similar when only including the core municipalities of the largest urban agglomerations in the Zurich region.

| ***Table A5.2.*** *Non-profit redevelopment: Displacement due to housing demolition or redevelopment* | | | | |
| --- | --- | --- | --- | --- |
|  | | | | |
|  | *Dependent variable:* | | | |
|  |  | | | |
|  | Distance to old location [log(km)] | Building built between 1945-1970 | Area per person [log(m2/P)] | Household income within 250m radius [median(CHF)] |
|  | *OLS* | *Logistic* | *OLS* | *OLS* |
|  | (1) | (2) | (3) | (4) |
|  | | | | |
| **Direct displacement** | **0.187** (0.066)** | **0.313 (0.298)** | **-0.036*** (0.009)** | **-0.081+ (0.046)** |
| Housing and neighborhood covariates | Yes | Yes | Yes | Yes |
| Individual-level covariates | Yes | Yes | Yes | Yes |
|  | | | | |
| Observations | 2,142 | 2,142 | 2,142 | 2,142 |
| R^2^ | 0.287 |  | 0.635 | 0.277 |
| Adjusted R^2^ | 0.243 |  | 0.612 | 0.232 |
| Log Likelihood |  | 1,001.397 |  |  |
| Akaike Inf. Crit. |  | 2,254.794 |  |  |
|  | | | | |
| *Note:* | Regression with all individual-level and housing unit covariates on housing location and characteristics after displacement. Sample includes all residents displaced from non-profit redevelopment projects in core municipalities of the Zurich region. Standard errors are clustered at the municipality level. ***p < 0.001, **p < 0.01, *p < 0.05, +p < 0.1 | | | |

## A5.3 Regression low-income residents (for-profit)

We find that, on average, it is mainly low-income residents who are displaced by demolition or renovation in the Zurich region. Their median household income is only 69.7% of the median household income of all residents who moved during the same period but for reasons other than housing demolition or renovation. Nevertheless, it could be that the movement patterns of those residents who earn significantly less than the median of all movers are different because they face even greater restrictions on where they can move to due to the scarcity of affordable housing in the Zurich region. We therefore restrict our sample to those residents who earned less than 60% of the median income of all residents who moved.

The results (Table A5.3) for the displacement location and housing characteristics of those low-income residents displaced from for-profit redevelopment projects remain robust compared to the main analysis (Table A3.1). This indicates that direct displacement has not only negative impacts for those that are most vulnerable to involuntary housing changes but also other residents.

| ***Table A5.3.*** *For-profit redevelopment: Displacement due to housing demolition or redevelopment* | | | | |
| --- | --- | --- | --- | --- |
|  | | | | |
|  | *Dependent variable:* | | | |
|  |  | | | |
|  | Distance to old location [log(km)] | Building built between 1945-1970 | Area per person [log(m2/P)] | Household income within 250m radius [median(CHF)] |
|  | *OLS* | *Logistic* | *OLS* | *OLS* |
|  | (1) | (2) | (3) | (4) |
|  | | | | |
| **Direct displacement** | **-0.189^**^ (0.069)** | **0.158^*^ (0.067)** | **-0.090^***^ (0.011)** | **-0.062 (0.038)** |
| Housing and neighborhood covariates | Yes | Yes | Yes | Yes |
| Individual-level covariates | Yes | Yes | Yes | Yes |
|  | | | | |
| Observations | 5,066 | 5,066 | 5,066 | 5,066 |
| R^2^ | 0.089 |  | 0.612 | 0.431 |
| Adjusted R^2^ | 0.057 |  | 0.599 | 0.411 |
| Log Likelihood |  | -2,702.080 |  |  |
| Akaike Inf. Crit. |  | 5,746.159 |  |  |
|  | | | | |
| *Note:* | Regression with all individual-level and housing unit covariates on housing location and characteristics after displacement. Sample includes all residents earning below 60% of the median household income of all residents who moved during the same time. Standard errors are clustered at the municipality level. ***p < 0.001, **p < 0.01, *p < 0.05, +p < 0.1 | | | |

## A5.4 Regression low-income residents (non-profit)

For those displaced from non-profit sites who earn less than 60% of the median household income of all residents who moved, we find a statistically significant association only for the distance measure. Displaced residents move further distances than similar residents who moved for reasons other than redevelopment. For all other dependent variables, we find positive but insignificant associations. This suggests that low-income residents living in non-profit housing are better protected when it comes to redevelopment. Cooperatives (non-profit investors) usually have to offer an alternative apartment and only if their members refuse, the residents have to find an alternative apartment on their own. Low-income residents are likely to accept this offer willingly, even if it means moving further away but staying in better quality housing. Together, the findings remain robust, showing that those displaced from non-profit redevelopment sites live in better housing situations after displacement.

| ***Table A5.4.*** *Non-profit redevelopment: Displacement due to housing demolition or redevelopment* | | | | |
| --- | --- | --- | --- | --- |
|  | | | | |
|  | *Dependent variable:* | | | |
|  |  | | | |
|  | Distance to old location [log(km)] | Building built between 1945-1970 | Area per person [log(m2/P)] | Household income within 250m radius [median(CHF)] |
|  | *OLS* | *Logistic* | *OLS* | *OLS* |
|  | (1) | (2) | (3) | (4) |
|  | | | | |
| **Direct displacement** | **0.331^***^ (0.086)** | **0.247 (0.317)** | **0.001 (0.010)** | **0.043 (0.038)** |
| Housing and neighborhood covariates | Yes | Yes | Yes | Yes |
| Individual-level covariates | Yes | Yes | Yes | Yes |
|  | | | | |
| Observations | 1,011 | 1,011 | 1,011 | 1,011 |
| R^2^ | 0.262 |  | 0.623 | 0.258 |
| Adjusted R^2^ | 0.174 |  | 0.579 | 0.170 |
| Log Likelihood |  | -465.388 |  |  |
| Akaike Inf. Crit. |  | 1,146.775 |  |  |
|  | | | | |
| *Note:* | Regression with all individual-level and housing unit covariates on housing location and characteristics after displacement. Sample includes all residents earning below 60% of the median household income of all residents who moved during the same time. Standard errors are clustered at the municipality level. ***p < 0.001, **p < 0.01, *p < 0.05, +p < 0.1 | | | |

## A5.5 Regression old buildings (for-profit)

In our main analysis (Table A3.1) we define old and poor quality buildings as those built in the post-war period between 1945 and 1970. We do so because these are buildings that were usually built in a fast manner and often have very small rooms. Additionally, they were not renovated for a long time (Heye, 2007). As a result, rents for such buildings are often comparatively low and it is common for developers to redevelop these sites, as a new and higher quality building can generate not only better housing quality but also higher profits.

However, it is likely that older dwellings are generally of lower quality and are more likely to be redeveloped or renovated, irrespective of a specific construction period. We therefore test whether displaced residents are generally more likely to move to old housing. To do so, we use two different variable specifications. First, we classify old buildings as those that were built before 1970 and second, we use a continuous variable for the age of the building. Our results shown in Table A5.5 remain very similar to the main regression results, as displaced residents from for-profit sites tend to move more often to older buildings in general.

| ***Table A5.5.*** *For-profit redevelopment: Displacement due to housing demolition or redevelopment* | | |
| --- | --- | --- |
|  | | |
|  | *Dependent variable:* | |
|  |  | |
|  | Building built before 1970 (dummy variable) | Building age (continuous variable) |
|  | *Logistic* | *OLS* |
|  | (1) | (2) |
|  | | |
| **Direct displacement** | **0.163^***^ (0.046)** | **3.559** (1.279)** |
| Housing and neighborhood covariates | Yes | Yes |
| Individual-level covariates | Yes | Yes |
|  | | |
| Observations | 13,138 | 12,872 |
| R^2^ |  | 0.093 |
| Adjusted R^2^ |  | 0.081 |
| Log Likelihood | -7,850.839 |  |
| Akaike Inf. Crit. | 16,057.680 |  |
|  | | |
| *Note:* | Regression with all individual-level and housing unit covariates on housing location and characteristics after displacement. The sample includes all residents displaced from for-profit redevelopment projects in the Zurich region. Standard errors are clustered at the municipality level. ***p < 0.001, **p < 0.01, *p < 0.05, +p < 0.1 | |

## A5.6 Regression old buildings (non-profit)

The same analysis as explained in A5.5 shows that there is no significant association between displacement from non-profit housing and moving to older buildings. The regression analysis focusing on displacement and building age (continuous variable) shows a negative and statistically significant association, meaning that those displaced from non-profit housing are less likely to move to older buildings than similar residents moving for reasons other than building demolition or renovation.

| ***Table A5.6.*** *Non-profit redevelopment: Displacement due to housing demolition or redevelopment* | | |
| --- | --- | --- |
|  | | |
|  | *Dependent variable:* | |
|  |  | |
|  | Building built before 1970 (dummy variable) | Building age (continuous variable) |
|  | *Logistic* | *OLS* |
|  | (1) | (2) |
|  | | |
| **Direct displacement** | **-0.149 (0.235)** | **-8.019** (2.974)** |
| Housing and neighborhood covariates | Yes | Yes |
| Individual-level covariates | Yes | Yes |
|  | | |
| Observations | 2,286 | 2,257 |
| R^2^ |  | 0.163 |
| Adjusted R^2^ |  | 0.110 |
| Log Likelihood | -1,263.098 |  |
| Akaike Inf. Crit. | 2,802.197 |  |
|  | | |
| *Note:* | Regression with all individual-level and housing unit covariates on housing location and characteristics after displacement. The sample includes all residents displaced from non-profit redevelopment projects in the Zurich region. Standard errors are clustered at the municipality level. ***p < 0.001, **p < 0.01, *p < 0.05, +p < 0.1 | |

## A5.7 Short-term residents

In this section, we focus on short-term residents. These are classified as residents who have lived in a building for one or two years before it is demolished or completely renovated. Since the introduction of the revised Spatial Planning Act in 2014 (SPA, 2012), the dynamics of temporary housing have gained momentum (Debrunner & Gerber, 2021). Today, there are various cooperatives and companies that rent out soon-to-be-demolished housing stock, usually through temporary leases and below-market rents. For a long time, most of this housing stock was rented out by cooperatives to specific groups (e.g. students or asylum seekers), and only recently has there been a shift towards the commodification of temporary housing (Debrunner & Gerber, 2021). Such apartments are usually rented out at below-market rents and are therefore mostly rented to students, low-income residents, and low-income migrants. Since those are specific groups of residents they likely differ from the long-term tenants, we present the summary tables and regression analysis of displaced short-term tenants separately from the main analysis where we focus exclusively on long-term residents. However, it is interesting to look at short-term tenants as they are likely to have particularly limited economic means to find accommodation.

### A5.7.1 Sample characteristics short-term residents

Table A5.7.1 shows the sample characteristics of all displaced residents. Column (1) shows the characteristics of long-term residents who had lived in a dwelling for three or more years before being displaced. In contrast, column (2) shows the characteristics of short-term residents who had lived in a dwelling for one or two years before it was demolished or completely renovated. Column (3) shows the characteristics of the PSM group, i.e. similar residents who had lived in a dwelling for up to two years and then moved for reasons other than demolition or renovation. Column (4) shows all residents who lived in a dwelling for up to two years and then moved to a new dwelling, and column (5) shows all residents living in the study area. Short-term residents are mostly low-income residents and non-Swiss citizens. The median income of displaced short-term residents lies at CHF 4,420 which is 65.3% of the median income of all movers. It is also slightly lower than the median income of long-term displaced residents, which lies at CHF 4,900. The share of short-term displaced residents who were born outside of Switzerland is 47.6% and only 57.5% hold a Swiss citizenship compared with 70.2% of all residents. Overall, short-term and long-term displaced residents are similarly different from the sample of all residents living in the study area.

However, important differences remain between the two groups of displaced persons. This is particularly evident in the category of residence permit: While the share of asylum seekers and refugees among the long-term displaced residents is 0.8%, the share of short-term displaced is 3.2%. As discussed above, the city and cooperatives work together to place asylum seekers in temporary accommodation (Debrunner & Gerber, 2021). In addition, the share of permit (B) holders is about twice as high among the short-term displaced compared to long-term displaced residents. Residence permit (B) is a five-year residence permit for EU or EFTA nationals residing in Switzerland, which is usually valid for five years and must be renewed if the resident wishes to stay longer. Taken together, the findings show that short-term displaced residents appear to be different from long-term displaced residents. As the research suggests, there are different reasons for who and why residents live in temporary accommodation, and therefore future research could analyse displacement patterns for different groups (such as students, asylum seekers and low-income residents) among those living in temporary housing.

| ***Table A5.7.1.*** *Sample characteristics short-term residents* | | | | | |
| --- | --- | --- | --- | --- | --- |
|  | **(1) Long-term displaced residents (N=7,712)** | **(2) Short-term displaced residents (N=4,257)** | **(3) Short-term comparison group (PSM) (N=4,257)** | **(4) All movers (N=146,090)** | **(5) All residents (N=1,277,306)** |
| **Household income**  **[CHF per month]** |  |  |  |  |  |
| Mean (SD) | 5,530 (3720) | 4,890 (3,420) | 5,010 (3,440) | 7,350 (4,120) | 7,330 (4,630) |
| Median | 4,900 | 4,420 | 4,600 | 6,770 | 6,540 |
| **Age** |  |  |  |  |  |
| Mean (SD) | 44.7 (17.6) | 38.7 (12.7) | 38.9 (13.3) | 37.4 (11.8) | 39.6 (19.0) |
| Median | 46.0 | 37.0 | 37.0 | 36.0 | 40.0 |
| **Sex** |  |  |  |  |  |
| Female | 3,699 (48.0%) | 1,863 (43.8%) | 1,849 (43.4%) | 71,172 (48.7%) | 624,316 (48.9%) |
| Male | 4,013 (52.0%) | 2,394 (56.2%) | 2,408 (56.6%) | 74,918 (51.3%) | 652,990 (51.1%) |
| **Nationality (continent)** |  |  |  |  |  |
| Africa | 136 (1.8%) | 231 (5.4%) | 204 (4.8%) | 1,916 (1.3%) | 13,585 (1.1%) |
| America (Central and South) | 120 (1.6%) | 68 (1.6%) | 69 (1.6%) | 1,553 (1.1%) | 10,203 (0.8%) |
| America (North) | 23 (0.3%) | 16 (0.4%) | 22 (0.5%) | 373 (0.3%) | 5,047 (0.4%) |
| Asia | 320 (4.1%) | 238 (5.6%) | 221 (5.2%) | 3,324 (2.3%) | 32,284 (2.5%) |
| EU and EFTA | 1,640 (21.3%) | 950 (22.3%) | 1057 (24.8%) | 28,379 (19.4%) | 246,159 (19.3%) |
| Europe (Outside EU) | 798 (10.3%) | 297 (7.0%) | 307 (7.2%) | 5,583 (3.8%) | 72,436 (5.7%) |
| Swiss | 4,675 (60.6%) | 2,449 (57.5%) | 2,374 (55.8%) | 104,921 (71.8%) | 897,218 (70.2%) |
| Without Nationality / No information | 0 (0%) | 8 (0.2%) | 3 (0.1%) | 41 (0.0%) | 374 (0.0%) |
| **Continent of birth** |  |  |  |  |  |
| Africa | 205 (2.7%) | 254 (6.0%) | 222 (5.2%) | 3,067 (2.1%) | 22,144 (1.7%) |
| America (Central and South) | 249 (3.2%) | 172 (4.0%) | 178 (4.2%) | 4,183 (2.9%) | 29,435 (2.3%) |
| America (North) | 42 (0.5%) | 23 (0.5%) | 29 (0.7%) | 1,124 (0.8%) | 10,921 (0.9%) |
| Asia | 497 (6.4%) | 332 (7.8%) | 333 (7.8%) | 5,860 (4.0%) | 56,726 (4.4%) |
| EU and EFTA | 1,479 (19.2%) | 811 (19.1%) | 910 (21.4%) | 25,402 (17.4%) | 224,542 (17.6%) |
| Europe (Outside EU) | 833 (10.8%) | 291 (6.8%) | 306 (7.2%) | 6,919 (4.7%) | 87,962 (6.9%) |
| Swiss | 3,975 (51.5%) | 2,231 (52.4%) | 2,116 (49.7%) | 96,899 (66.3%) | 817,090 (64.0%) |
| Without Nationality / No information | 432 (5.6%) | 143 (3.4%) | 163 (3.8%) | 2,636 (1.8%) | 28,486 (2.2%) |
| **Resident permit** |  |  |  |  |  |
| Asylum seeker / Refugee status | 63 (0.8%) | 138 (3.2%) | 108 (2.5%) | 1,115 (0.8%) | 5,164 (0.4%) |
| Other | 6 (0.1%) | 21 (0.5%) | 17 (0.4%) | 240 (0.2%) | 4,462 (0.3%) |
| Residence permit (B) | 734 (9.5%) | 831 (19.5%) | 901 (21.2%) | 17,236 (11.8%) | 147,751 (11.6%) |
| Settlement permit (C) | 2,234 (29.0%) | 818 (19.2%) | 857 (20.1%) | 22,578 (15.5%) | 222,711 (17.4%) |
| This table shows the socio-demographic characteristics of long-term displaced residents (column 1) and short-term residents who moved because of housing demolition or renovation (column 2). Column (3) shows the PSM comparison group of all short-term displaced residents. Additionally, it shows the characteristics of all residents who had been living in an apartment for up to two years and then moved to a new apartment for reasons other than redevelopment (column 4). Finally, column (5) shows all residents of the Zurich and Winterthur agglomeration. Outliers and observations of households with more than 10 members were excluded from all samples. Short term residents are defined as those residents who had been living for one or two years at the old location (before demolition or complete renovation) and then moved to a new apartment. | | | | | |

### A5.7.2 Housing characteristics short-term residents

Table A5.7.2 shows the housing characteristics of long-term and short-term residents who were living in a building before redevelopment took place. Column (1) shows the housing characteristics of long-term residents who were living in for-profit housing. Column (2) shows the housing characteristics of displaced short-term residents from for-profit sites and column (3) shows the respective comparison group. Similarly, column (4) shows long-term displaced residents from non-profit housing and columns (5 and 6) show short-term displaced residents and the respective comparison group of those who had been living in non-profit housing. On average, displaced short-term residents do not improve their housing situation after displacement in terms of square meters per person. Nevertheless, compared to their before housing situation, short-term displaced residents move more often to newer housing and less often to buildings built before 1970 respectively. In comparison to long-term residents, however, they move less often to buildings built after 1970. On average, they live closer to the city center both before and after displacement compared to displaced long-term residents. As suggested by Debrunner and Gerber (2021), temporary housing can be seen as an affordable housing option for students who want to live close to the city center but also for low-income residents who need to live close to the city center because of their workplace and cannot afford to move to a more stable housing situation. In such cases, temporary housing can mean that vulnerable groups of residents are forced to move into insecure housing.

| ***Table A5.7.2.*** *Housing characteristics short-term residents* | | | | | | |
| --- | --- | --- | --- | --- | --- | --- |
|  | **(1) Long-term displaced for-profit (N=6,569)** | **(2) Short-term displaced for-profit (N=3,403)** | **(3) Comparison group short-term displaced for-profit (PSM) (N=3,403)** | **(4) Long-term displaced non-profit (N=1,143)** | **(5) Short-term displaced non-profit (N=854)** | **(6) Comparison group short-term displaced non-profit (PSM) (N=854)** |
| **Investor type after** |  |  |  |  |  |  |
| For-profit | 5,929 (90.3%) | 3,097 (91.0%) | 3,150 (92.6%) | 583 (51.0%) | 532 (62.3%) | 557 (65.2%) |
| Non-profit | 640 (9.7%) | 306 (9.0%) | 253 (7.4%) | 560 (49.0%) | 322 (37.7%) | 297 (34.8%) |
| **Square meters apartment**  **before** |  |  |  |  |  |  |
| Mean (SD) | 77.7 (26.6) | 75.2 (28.5) | 87.0 (37.5) | 67.6 (14.9) | 64.9 (13.6) | 80.3 (34.3) |
| **Square meters apartment**  **after** |  |  |  |  |  |  |
| Mean (SD) | 88.2 (33.7) | 83.7 (38.0) | 92.7 (42.4) | 83.3 (28.6) | 78.9 (35.3) | 87.8 (37.1) |
| **Household size before** |  |  |  |  |  |  |
| Mean (SD) | 2.91 (1.52) | 2.39 (1.31) | 2.67 (1.45) | 2.69 (1.36) | 2.17 (1.12) | 2.93 (1.52) |
| **Household size after** |  |  |  |  |  |  |
| Mean (SD) | 2.93 (1.57) | 2.61 (1.47) | 2.69 (1.45) | 2.73 (1.38) | 2.44 (1.35) | 2.84 (1.61) |
| **Square meters per person before** |  |  |  |  |  |  |
| Mean (SD) | 34.1 (20.0) | 38.8 (20.7) | 38.7 (19.8) | 31.8 (16.4) | 37.1 (17.8) | 32.7 (16.8) |
| **Square meters per person after** |  |  |  |  |  |  |
| Mean (SD) | 37.5 (21.8) | 38.6 (21.6) | 41.0 (22.3) | 36.8 (18.3) | 38.7 (20.3) | 37.4 (19.9) |
| **Building construction year before** |  |  |  |  |  |  |
| <1945 | 2,307 (35.1%) | 1,395 (41.0%) | 978 (28.7%) | 386 (33.8%) | 336 (39.3%) | 198 (23.2%) |
| 1945-1970 | 3231 (49.2%) | 1,523 (44.8%) | 939 (27.6%) | 635 (55.6%) | 466 (54.6%) | 321 (37.6%) |
| >1970 | 1031 (15.7%) | 485 (14.3%) | 1,486 (43.7%) | 122 (10.7%) | 52 (6.1%) | 335 (39.2%) |
| **Building construction year after** |  |  |  |  |  |  |
| <1945 | 1326 (20.2%) | 1,166 (34.3%) | 875 (25.7%) | 213 (18.6%) | 296 (34.7%) | 245 (28.7%) |
| 1945-1970 | 2078 (31.6%) | 1,040 (30.6%) | 823 (24.2%) | 386 (33.8%) | 263 (30.8%) | 215 (25.2%) |
| >1970 | 3165 (48.2%) | 1,197 (35.2%) | 1,705 (50.1%) | 544 (47.6%) | 295 (34.5%) | 394 (46.1%) |
| **Distance between old and new apartment in km** |  |  |  |  |  |  |
| Mean (SD) | 3.27 (4.64) | 4.19 (5.47) | 5.57 (6.59) | 2.84 (4.27) | 3.95 (5.00) | 4.64 (5.66) |
| **Distance to center before** |  |  |  |  |  |  |
| Mean (SD) | 7.46 (5.78) | 6.15 (5.29) | 8.49 (6.14) | 4.34 (2.84) | 3.97 (2.72) | 5.40 (4.21) |
| **Distance to center after** |  |  |  |  |  |  |
| Mean (SD) | 8.38 (5.83) | 6.93 (5.62) | 8.70 (6.23) | 5.13 (3.61) | 4.81 (3.96) | 6.00 (4.88) |
| **Median household income in CHF before (250m radius)** |  |  |  |  |  |  |
| Mean (SD) | 6,500 (1400) | 6,710 (1,410) | 6,540 (1,460) | 5,960 (919) | 5,900 (926) | 5,990 (967) |
| **Median household income in CHF after (250m radius)** |  |  |  |  |  |  |
| Mean (SD) | 6,650 (1220) | 6,840 (1280) | 6,790 (1,260) | 6,420 (1050) | 6,600 (1,200) | 6,620 (1,090) |
| This table shows the housing characteristics of residents displaced long-term residents from for-profit housing (column 1) and non-profit housing (column 4). In contrast, columns 2 and 5 show all residents who had been living for only one or two years in an apartment before displacement. Columns 3 and 6 show the respective PSM comparison group of short-term residents displaced from for-profit and non-profit housing. | | | | | | |

### A5.7.3 Regression analyses short-term residents

Finally, we run the same OLS regression models as in our main analysis, but this time focusing on short-term residents. Tables A5.7.3 and A5.7.4 show the results for short-term residents displaced from for-profit and non-profit redevelopment projects respectively. For short-term residents displaced from for-profit housing, we find no significant association between being displaced and the relocation distance. However, short-term displaced residents from for-profit redevelopment projects are more likely to move to buildings built between 1945-1970 and they are more likely to relocate to smaller apartments and lower-income neighborhoods. For short-term residents displaced from non-profit housing, we find a statistically significant association between displacement and moving to apartments with less area per person.

| ***Table A5.7.3.*** *For-profit redevelopment: Displacement due to housing demolition or redevelopment* | | | | |
| --- | --- | --- | --- | --- |
|  | | | | |
|  | *Dependent variable:* | | | |
|  |  | | | |
|  | Distance to old location [log(km)] | Building built between 1945-1970 | Area per person [log(m2/P)] | Household income within 250m radius [median(CHF)] |
|  | *OLS* | *Logistic* | *OLS* | *OLS* |
|  | (1) | (2) | (3) | (4) |
|  | | | | |
| **Direct displacement** | **-0.154 (0.121)** | **0.152^*^ (0.076)** | **-0.036^**^ (0.011)** | **-0.053^*^ (0.023)** |
| Housing and neighborhood covariates | Yes | Yes | Yes | Yes |
| Individual-level covariates | Yes | Yes | Yes | Yes |
|  | | | | |
| Observations | 6,806 | 6,806 | 6,806 | 6,806 |
| R^2^ | 0.085 |  | 0.504 | 0.396 |
| Adjusted R^2^ | 0.061 |  | 0.491 | 0.380 |
| Log Likelihood |  | -3,447.980 |  |  |
| Akaike Inf. Crit. |  | 7,245.960 |  |  |
|  | | | | |
| *Note:* | Regression with all individual-level and housing unit covariates on housing location and characteristics after displacement. The sample includes all short-term residents displaced from for-profit redevelopment projects in the Zurich region. Standard errors are clustered at the municipality level. ***p < 0.001, **p < 0.01, *p < 0.05, +p < 0.1 | | | |

| ***Table A5.7.4.*** *Non-profit redevelopment: Displacement due to housing demolition or redevelopment* | | | | |
| --- | --- | --- | --- | --- |
|  | | | | |
|  | *Dependent variable:* | | | |
|  |  | | | |
|  | Distance to old location [log(km)] | Building built between 1945-1970 | Area per person [log(m2/P)] | Household income within 250m radius [median(CHF)] |
|  | *OLS* | *Logistic* | *OLS* | *OLS* |
|  | (1) | (2) | (3) | (4) |
|  | | | | |
| **Direct displacement** | **-0.178 (0.119)** | **0.184 (0.143)** | **-0.026^**^ (0.009)** | **-0.047 (0.086)** |
| Housing and neighborhood covariates | Yes | Yes | Yes | Yes |
| Individual-level covariates | Yes | Yes | Yes | Yes |
|  | | | | |
| Observations | 1,708 | 1,708 | 1,708 | 1,708 |
| R^2^ | 0.210 |  | 0.577 | 0.262 |
| Adjusted R^2^ | 0.145 |  | 0.542 | 0.201 |
| Log Likelihood |  | -835.096 |  |  |
| Akaike Inf. Crit. |  | 1,932.192 |  |  |
|  | | | | |
| *Note:* | Regression with all individual-level and housing unit covariates on housing location and characteristics after displacement. The sample includes all short-term residents displaced from non-profit redevelopment projects in the Zurich region. Standard errors are clustered at the municipality level. ***p < 0.001, **p < 0.01, *p < 0.05, +p < 0.1 | | | |

## A5.8 Distance to center

As an additional analysis, and to see whether displaced residents tend to move from the center of the city towards suburban areas, we run additional analyses based on the distance to the center and on how far residents move.

### A5.8.1 Housing characteristics and relocation distance

Table 2 in the main text shows that displaced residents consume more floor space after displacement. In this section we distinguish between those residents who move less than one kilometer and those who move further away. Table 5.8.1 shows the housing characteristics based on how far residents moved. When moving to buildings further away, the floor space consumption of those displaced from for-profit sites increases more (increase by 12.6%) compared to residents who stay within one kilometer to their old home (increase by 6.1%). For those who move from non-profit sites the floor space increases by 15.1% when residents move far away and when they stay close to their old homes 16.3% respectively. Additionally, we see that all displaced residents who move more than 1km move slightly less often to older buildings compared to those who stay within a one-kilometer radius of their old home. Compared to their previous housing situation, all displaced residents move less often to old buildings. With regard to the income level of the place where the displaced residents move, we do not find large differences depending on the distance of the move.

***Table A5.8.1.*** *Housing characteristics depending on relocation distance*

|  | **Displaced for-profit** | | **Comparison group for-profit (PSM)** | | **Displaced non-profit** | | **Comparison group non-profit (PSM)** | |
| --- | --- | --- | --- | --- | --- | --- | --- | --- |
|  | **move more than 1km (N=3,859)** | **stay within 1km (N=2,710)** | **move more than 1km (N=4,419)** | **stay within 1km (N=2,150)** | **move more than 1km (N=630)** | **stay within 1km (N=513)** | **move more than 1km (N=622)** | **stay within 1km (N=521)** |
| **Investor Type after** |  |  |  |  |  |  |  |  |
| For-profit | 3,443 (89.2%) | 2,486 (91.7%) | 4,067 (92.0%) | 1,991 (92.6%) | 382 (60.6%) | 201 (39.2%) | 488 (78.5%) | 161 (30.9%) |
| Non-profit | 416 (10.8%) | 224 (8.3%) | 352 (8.0%) | 159 (7.4%) | 248 (39.4%) | 312 (60.8%) | 134 (21.5%) | 360 (69.1%) |
| **Square meters apartment before** |  |  |  |  |  |  |  |  |
| Mean (SD) | 75.8 (25.9) | 80.5 (27.2) | 96.6 (43.2) | 94.5 (37.1) | 66.5 (16.3) | 68.9 (12.8) | 85.7 (27.6) | 83.1 (24.5) |
| **Square meters apartment after** |  |  |  |  |  |  |  |  |
| Mean (SD) | 87.8 (34.4) | 88.7 (32.7) | 101 (43.7) | 100 (39.3) | 83.6 (31.3) | 82.8 (25.0) | 96.7 (38.3) | 89.5 (27.6) |
| **Household size before** |  |  |  |  |  |  |  |  |
| Mean (SD) | 2.81 (1.52) | 3.07 (1.50) | 2.94 (1.45) | 3.30 (1.43) | 2.53 (1.40) | 2.87 (1.27) | 3.19 (1.45) | 3.31 (1.42) |
| **Household size after** |  |  |  |  |  |  |  |  |
| Mean (SD) | 2.81 (1.57) | 3.10 (1.57) | 2.77 (1.46) | 3.06 (1.48) | 2.63 (1.46) | 2.86 (1.27) | 2.88 (1.50) | 3.11 (1.51) |
| **Square meters per person before** |  |  |  |  |  |  |  |  |
| Mean (SD) | 34.8 (20.4) | 33.0 (19.3) | 39.4 (21.8) | 34.3 (20.1) | 33.7 (17.0) | 29.5 (15.2) | 32.3 (16.6) | 30.1 (16.3) |
| **Square meters per person after** |  |  |  |  |  |  |  |  |
| Mean (SD) | 39.2 (23.2) | 35.0 (19.4) | 43.6 (23.7) | 39.7 (22.9) | 38.8 (19.8) | 34.3 (15.9) | 39.6 (20.3) | 34.5 (16.5) |
| **Building construction year before** |  |  |  |  |  |  |  |  |
| <1945 | 1,463 (37.9%) | 844 (31.1%) | 1,166 (26.4%) | 521 (24.2%) | 213 (33.8%) | 173 (33.7%) | 116 (18.6%) | 113 (21.7%) |
| 1945-1970 | 1,813 (47.0%) | 1,418 (52.3%) | 1,285 (29.1%) | 665 (30.9%) | 355 (56.3%) | 280 (54.6%) | 262 (42.1%) | 203 (39.0%) |
| >1970 | 583 (15.1%) | 448 (16.5%) | 1,968 (44.5%) | 964 (44.8%) | 62 (9.8%) | 60 (11.7%) | 244 (39.2%) | 205 (39.3%) |
| **Building construction year after** |  |  |  |  |  |  |  |  |
| <1945 | 780 (20.2%) | 546 (20.1%) | 859 (19.4%) | 424 (19.7%) | 115 (18.3%) | 98 (19.1%) | 116 (18.6%) | 100 (19.2%) |
| 1945-1970 | 1,174 (30.4%) | 904 (33.4%) | 931 (21.1%) | 445 (20.7%) | 207 (32.9%) | 179 (34.9%) | 123 (19.8%) | 155 (29.8%) |
| >1970 | 1,905 (49.4%) | 1,260 (46.5%) | 2,629 (59.5%) | 1,281 (59.6%) | 308 (48.9%) | 236 (46.0%) | 383 (61.6%) | 266 (51.1%) |
| **Distance between old and new apartment in km** |  |  |  |  |  |  |  |  |
| Mean (SD) | 5.28 (5.18) | 0.420 (0.287) | 7.56 (6.86) | 0.402 (0.291) | 4.90 (4.86) | 0.316 (0.285) | 7.29 (6.37) | 0.303 (0.286) |
| **Distance to center before** |  |  |  |  |  |  |  |  |
| Mean (SD) | 6.79 (5.67) | 8.43 (5.81) | 8.76 (6.15) | 9.71 (6.12) | 4.04 (2.56) | 4.71 (3.11) | 5.63 (4.00) | 5.77 (4.47) |
| **Distance to center after** |  |  |  |  |  |  |  |  |
| Mean (SD) | 8.33 (5.83) | 8.46 (5.82) | 9.50 (6.31) | 9.71 (6.12) | 5.48 (3.94) | 4.71 (3.12) | 7.77 (5.49) | 5.77 (4.49) |
| **Median household income in CHF before (250m radius)** |  |  |  |  |  |  |  |  |
| Mean (SD) | 6,540 (1410) | 6,440 (1380) | 6,580 (1530) | 6,460 (1370) | 5,800 (884) | 6,170 (922) | 5,840 (1080) | 5,950 (949) |
| **Median household income in CHF after (250m radius)** |  |  |  |  |  |  |  |  |
| Mean (SD) | 6,680 (1210) | 6,600 (1240) | 6,870 (1300) | 6,670 (1230) | 6,400 (1080) | 6,450 (1020) | 6,690 (1120) | 6,350 (921) |

### A5.8.2 Analyses distance to center

Finally, we examine whether displaced residents move further away from the city center and towards the suburbs compared to the PSM comparison group. To do so, we calculate for each resident the distance between their housing location after relocation and the urban center, measured as the distance between coordinates of the building where residents live and the coordinates of the train station of the City of Zurich and Winterthur. We then select the minimal distance.

Regression model (1) shows the result for those displaced from for-profit redevelopment projects. We do not find a statistically significant association between the distance to the center and being directly displaced. In contrast, model (2) shows the results for those displaced from non-profit redevelopment sites. Here, we find that being displaced is associated with moving to a building further away from the urban center.

| ***Table A5.8.2.1.*** *Redevelopment: Displacement due to housing demolition or redevelopment* | | |
| --- | --- | --- |
|  | | |
|  | *Dependent variable:* | |
|  |  | |
|  | Distance to center [log(km)] (for-profit) | Distance to center [log(km)] (non-profit) |
|  | *OLS* | *OLS* |
|  | (1) | (2) |
|  | | |
| **Direct displacement** | **-0.002 (0.006)** | **0.019^***^ (0.005)** |
| Housing and neighborhood covariates | Yes | Yes |
| Individual-level covariates | Yes | Yes |
|  | | |
| Observations | 13,138 | 2,286 |
| R^2^ | 0.847 | 0.749 |
| Adjusted R^2^ | 0.845 | 0.733 |
|  | | |
| *Note:* | Regression with all individual-level and housing unit covariates on housing location and characteristics after displacement. Model (1) includes all residents displaced from for-profit redevelopment projects in the Zurich region. Model (2) includes all residents displaced from non-profit redevelopment projects in the Zurich region. Standard errors are clustered at the municipality level. ***p < 0.001, **p < 0.01, *p < 0.05, +p < 0.1 | |

In a last step we run the regression models using the difference between the distance to the urban center after and before displacement as dependent variable. The variable ranges from -25.9km to 24km, negative numbers indicating that residents moved closer to the urban center and positive numbers indicate they moved further away. For both residents displaced from for-profit sites (1) and those displaced from non-profit (2) redevelopment projects we see a positive and significant association between being displaced and an increase in distance to the urban center.

| ***Table A5.8.2.2.*** *Redevelopment: Displacement due to housing demolition or redevelopment* | | |
| --- | --- | --- |
|  | | |
|  | *Dependent variable:* | |
|  |  | |
|  | Difference distance to center after and before relocation [km] (for-profit) | Difference distance to center after and before relocation [km] (non-profit) |
|  | *OLS* | *OLS* |
|  | (1) | (2) |
|  | | |
| Direct displacement | 1.018^***^ (0.216) | 0.722^*^ (0.302) |
| Housing and neighborhood covariates | Yes | Yes |
| Individual-level covariates | Yes | Yes |
|  | | |
| Observations | 13,138 | 2,286 |
| R^2^ | 0.187 | 0.544 |
| Adjusted R^2^ | 0.176 | 0.515 |
|  | | |
| *Note:* | Regression with all individual-level and housing unit covariates on housing location and characteristics after displacement. Model (1) includes all residents displaced from for-profit redevelopment projects in the Zurich region. Model (2) includes all residents displaced from non-profit redevelopment projects in the Zurich region. Standard errors are clustered at the municipality level. ***p < 0.001, **p < 0.01, *p < 0.05, +p < 0.1 | |

# A6 References

Aratani, Y. (2011). Socio-demographic Variations of Homeowners and Differential Effects of Parental Homeownership on Offspring’s Housing Tenure. *Housing Studies*, *26*(5), 723–746. https://doi.org/10.1080/02673037.2011.581912

Carlson, D., Haveman, R., Kaplan, T., & Wolfe, B. (2012). Long-term earnings and employment effects of housing voucher receipt. *Journal of Urban Economics*, *71*(1), 128–150. https://doi.org/10.1016/j.jue.2011.07.001

Cunningham, S. (2021). *Causal inference: The mixtape*. Yale University Press.

Debrunner, G., & Gerber, J.-D. (2021). The commodification of temporary housing. *Cities*, *108*, 102998. https://doi.org/10.1016/j.cities.2020.102998

Desmond, M., & Kimbro, R. T. (2015). Eviction’s Fallout: Housing, Hardship, and Health. *Social Forces*, *94*(1), 295–324. https://doi.org/10.1093/sf/sov044

Heye, C. (2007). *Sozialräumliche Prozesse in urbanen Räumen der Schweiz* [University of Zurich]. https://www.zora.uzh.ch/id/eprint/163814/1/20090531_002264227.pdf

SPA. (2012). *The Federal Spatial Planning Act*. https://www.are.admin.ch/are/de/home/raumentwicklung-und-raumplanung/raumplanungsrecht/revision-des-raumplanungsgesetzes--rpg-/rpg1.html

VanderWeele, T. J. (2019). Principles of confounder selection. *European Journal of Epidemiology*, *34*(3), 211–219. https://doi.org/10.1007/s10654-019-00494-6
